# A Comprehensive View on the Molecular Mechanism of the Diels–Alder Reaction Between 2,3-Dimethylbutadiene and E-2-Phenyl-1-Nitroethene: CDFT/ELF, ^14^C Kinetic Isotope Effects, Electron Density Transfers, Reaction Profiles and BET Analyses

**DOI:** 10.3390/ijms27209223

**Published:** 2026-10-17

**Authors:** Przemysław Woliński, Weronika Łącka, Radomir Jasiński

**Affiliations:** Cracow University of Technology, Faculty of Chemical Engineering and Technology, Warszawska 24, 31-155 Cracow, Polandweronika.lacka@student.pk.edu.pl (W.Ł.)

**Keywords:** Diels–Alder reaction, mechanism, geometry of transition state, kinetic isotope effects

## Abstract

A comprehensive theoretical study of the model Diels–Alder reaction between 2,3-dimethylbutadiene and E-2-phenyl-1-nitroethene was performed. This work uniquely combines experimentally measured kinetic isotope effects with DFT calculations. The results not only provide insight into the nature of the reaction under study but also provide information on how well different levels of theory can predict the key geometric parameters of the transition states of Diels–Alder reactions with the participation of carbodienes and conjugated nitroalkenes.

## 1. Introduction

From its discovery [1] to this day, the Diels–Alder reaction (formally (4 + 2) cycloaddition) has been the most universal method for the efficient and selective synthesis of organic compounds containing a 6-membered carbocyclic segment [2,3,4,5]. Views on the factors determining its course and the molecular mechanism have undergone profound evolution during this time. Just a few decades ago, the pericyclic, “concerted” mechanism with a synchronous, circular displacement of three electron pairs within a cyclic transition state bearing the hallmarks of an aromatic structure was accepted as axiomatic [6]. A similar mechanism was also postulated for (3 + 2) cycloaddition reactions [7]. However, later cases were found that eluded this idealistic definition. It turned out that in some cases, stepwise mechanisms, with a diradical [8] or zwitterionic intermediate [9,10,11], can compete with the single-step mechanism. Recent research using BET computational techniques has indicated that even single-step reactions should not necessarily be considered pericyclic, as they essentially constitute a sequence of elementary transformations differing in the manner and direction of electron density redistribution along the reaction coordinate [12].

Many techniques currently exist for studying the mechanisms of cycloaddition reactions. The most valuable ones involve determining the specific physical properties of transition states using indirect methods. For example, with experimentally measured rate constants at various temperatures, activation enthalpies and entropies can be determined [13,14,15]. Hammet correlations [16,17,18] define the mode of transfer of electronic effects to reaction centers and allow for the diagnosis of the direction and magnitude of charge transfer between substructures [19]. Correlations with *Dimroth* constants, on the other hand, allow for confirming or ruling out the existence of ionic intermediates [20,21,22]. An exceptionally valuable, although rarely used in practice, technique is the measurement of kinetic isotope effects (KIEs) [23,24]. In an elementary cycloaddition step, as the reacting system transitions to the activated complex, the hybridization of the carbon atoms involved in forming new bonds changes. Consequently, the frequencies of out-of-plane bending vibrations associated with these carbon atoms increase. These vibrational frequencies are related to zero-point vibrational energy. Substituting a heavier isotope at the C12 reaction center alters the difference in zero-point vibrational energy between the initial state and the activated complex. The observed change corresponds to the ratio of rate constants for reactions involving unlabeled versus isotopically labeled reactants. This phenomenon is known as the kinetic isotope effect. SKIE analysis can be effectively employed in mechanistic studies. Since the degree of rehybridization at the reaction centers is closely linked to the extent of the formation of new σ-bonds, the structure of the transition state can be inferred through the analysis of secondary isotope effects. The connection between the experimental KIEs and the reported C–C bond distances is somewhat indirect. So, formally, the bond lengths are inferred from the DFT level that reproduces the experimental KIEs well, rather than obtained directly from the KIE values.

**Scheme 1:**
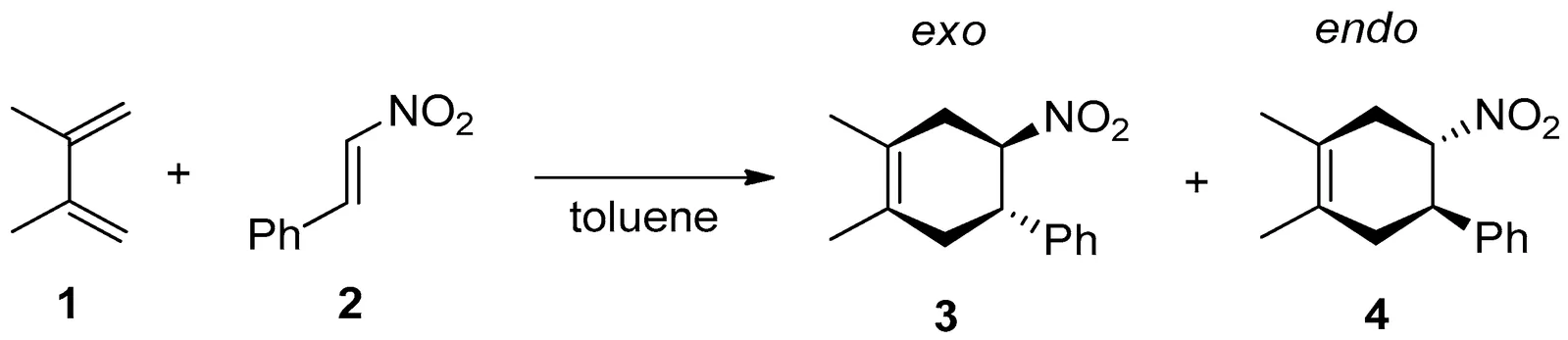
Reaction of 2,3-dimethylbuta-1,3-diene **1** with (E)-2-phenyl-1-nitroethene **2**.

Some time ago, *Kupczyk-Subotkowska* and colleagues [25] experimentally measured KIEs for a model reaction between 2,3-dimethylbutadiene (**1**) and (*E*)-2-phenyl-1-nitroethene (**2**) (Scheme 1). We decided to treat this process as a model to verify typical DFT levels recently used to explore the mechanistic aspects of cycloaddition reactions. We supplemented these studies with an analysis of intermolecular interactions in the framework of Conceptual Density Functional Theory [26,27] and a full description of the reaction energy profiles.

We hope that the obtained results will not only allow for a better understanding of the mechanism of cycloaddition involving carbodienes and conjugated nitroalkenes but also provide a basis for the better selection of DFT techniques for mechanistic studies.

## 2. Results and Discussion

In the first stage of our work, we decided to analyze the electronic structures and global and local electronic properties of the reaction components based on Conceptual Density Functional Theory (CDFT) indices [26,27]. For this purpose, we used structures optimized at the ωB97X-D/6-311G(d) level of theory. We chose this DFT algorithm to enable direct comparisons with analogous electronic parameters of other unsaturated molecular segments and nitro compounds [28,29,30], which have recently been examined based on ωB97X-D/6-311G(d) calculations. The results obtained are summarized in Table 1.

A comparison of the chemical electron potentials (μ) of the reactants suggests that in the elementary reaction, electron density should flow toward the nitroalkene substructure. This prediction is consistent with empirical parameters determined from *Hammet* correlations for cycloadditions involving nitroalkenes and nucleophilic partners [18]. At the same time, the global electrophilicity index value classifies the diene as a weak electrophilic reagent, with a high global nucleophilicity index (N). On the other hand, the expected electron density acceptor **2** should be classified as a strong electrophile (see ω value) on the universal reactivity scale [31]. Importantly, the above parameters classify the title process as moderately polar [32].

**Figure 1:**
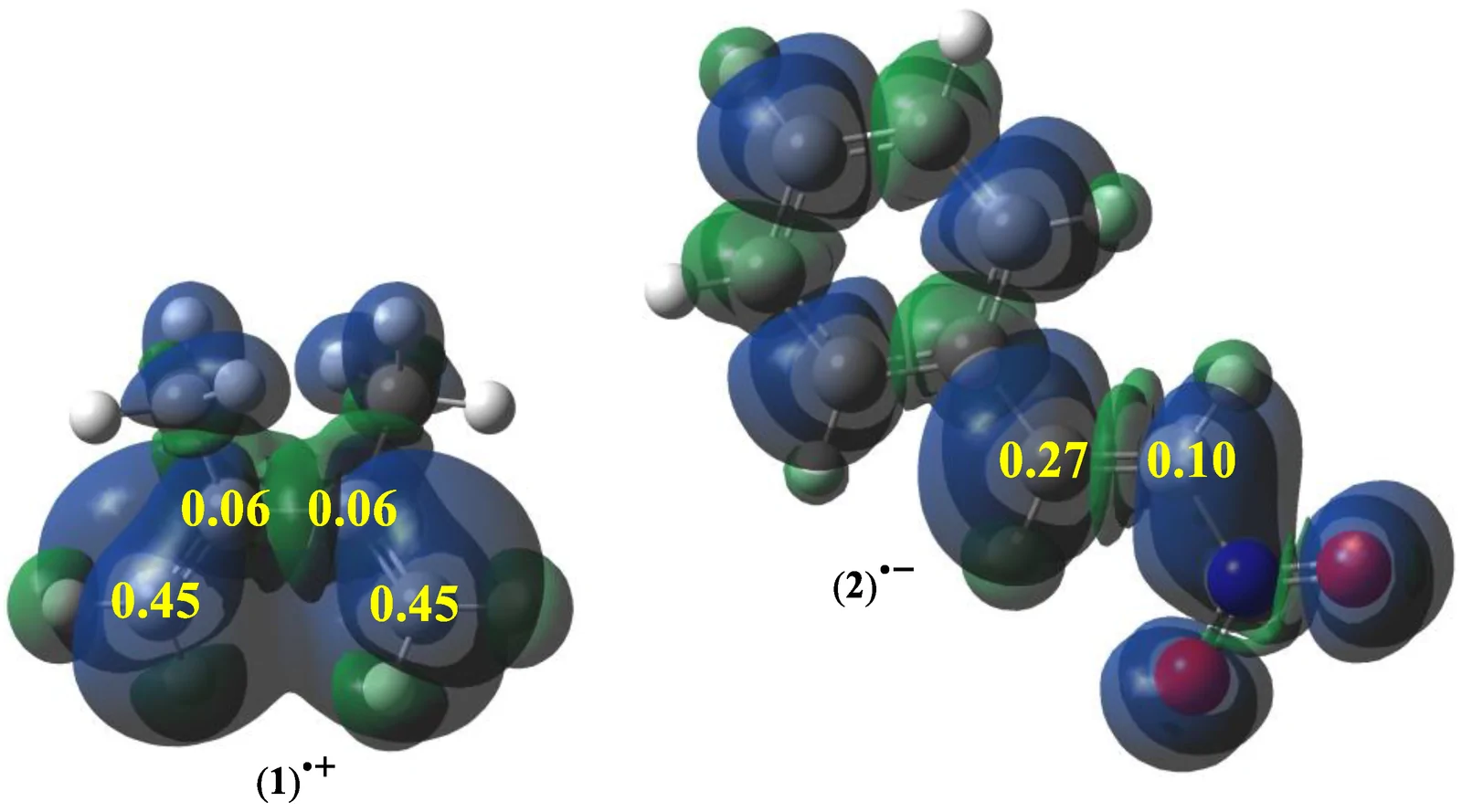
Local reactivities of 2,3-dimethylbutadiene **1** and E-2-phenyl-1-nitroethene **2**.

Global CDFT indices allow for a general diagnosis of the reactivity types of individual reaction components. However, these activities are not uniformly distributed throughout the molecules. In particular, in the case of diene **1**, the terminal positions of the >C=C-C=C< fragment are clearly more strongly nucleophilic. This is indicated by the values of the corresponding Parr spin density indices (Figure 1). In the case of nitroalkene, the b-carbon atom of the nitrovinyl segment is more electrophilic, as evidenced by the values of the corresponding Parr spin density indices (Figure 1). The local reactivity of nitroalkene **2** follows a trend typical for other conjugated nitrovinyls, such as nitroethene [33], 1-chloronitroethene [34], 2-phosphorylated nitroethenes [35], 2-CX_3_-nitroethenes [36] and conjugated nitrodienes [37,38]. An analysis of the local reactivity distribution indicates that the process should be determined by the attack of the nucleophilic, terminal carbon atom of the diene on the electrophilically activated 2-position of the nitrovinyl segment of the nitroalkene. Consequently, this is where a new chemical bond should form more rapidly. It should be emphasized here that CDFT indices describe molecules in the gas phase, so they should be taken with some caution when interpreting real reactions in a solvent.

**Figure 2:**
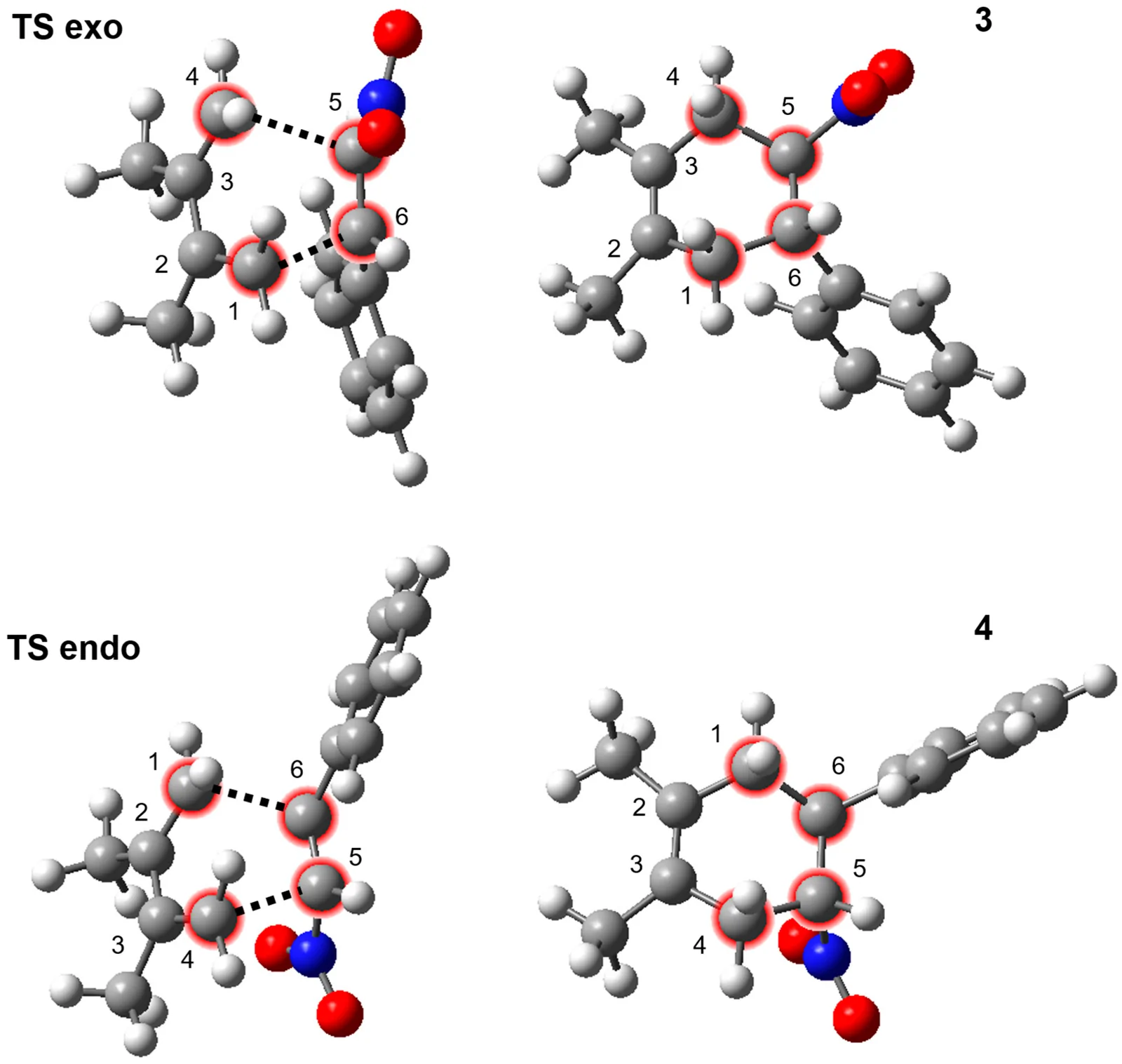
Views of critical structures of Diels–Alder reaction between 2,3-dimethylbutadiene **1** and E-2-phenyl-1-nitroethene **2** in toluene solution in light of ωB97X-D/6-311G(d) (PCM) calculations.

An exploration of the energy profiles of both stereoisomeric reaction channels leading to exo- and endo-nitro cycloadducts **3** and **4** revealed that in both cases there is only one transition state between the substrate minimum and the product minima (Table 2, Table 3 and Table 4). This was clearly confirmed by an analysis of the IRC trajectories. All attempts to identify diradical or zwitterionic intermediates along the pathways of the process under investigation were unsuccessful. Furthermore, there is no evidence for the formation of diradicals or zwitterions with an “extended” conformation, which could theoretically exist alongside the cycloaddition pathways. The nature of the localized saddle points is further confirmed by vibrational analysis. The structures of these transition states are illustrated in Figure 2. As can be seen, they are biplanar in nature, characteristic of the “carbo” Diels–Alder reaction [39,40,41]. Within them, two new bonds form between the C4-C5 and C6-C1 atoms. However, the degree of bond advancement varies. In particular, the C6-C1 bond always forms faster, as expected from the CFDT analysis. Table 2 summarizes data on key interatomic distances calculated at different levels of theory. As can be seen, depending on the algorithm used, the asynchrony of TSs can be predicted over a fairly wide range. Which of these results will be the closest to the true value? The answer to this question comes from the analysis of kinetic isotope effects. These parameters, as is known, are directly proportional to the degree of rehybridization at the reaction centers and, consequently, to the lengths of the newly formed chemical bonds. The authors of [25] managed to measure these effects experimentally, providing an excellent foundation for the search for a level of theory that accurately reflects the geometric nature of the transition states. It turned out that the satisfactory fits of the KIE values can be achieved at the ωB97X-D/6-311G(d) level of theory. This means that the lengths of the key interatomic distances between C4-C5 and C6-C1 should be determined for the exo- and endo-nitro transition states as 2.347 Å and 2.136 Å and 2.376 Å and 2.116 Å (for TSexo and TSendo, respectively). The asynchronous nature of TSs is accompanied by a non-uniform electron density transfer process. The GEDT index, which illustrates this effect, has a value of 0.18 e at this level of theory, which corresponds to a moderately polar but not zwitterionic reaction.

An analysis of the activation parameters summarized in Table 4 provides important information. As expected, dispersion corrections underestimate the activation enthalpy values. This effect has been previously observed for Diels–Alder reactions [42,43,44,45]. However, in general, the activation enthalpy values are not very large and remain within the range typical of single-step reactions [46,47,48,49], in which the energy changes resulting from bond breaking are compensated by the energy changes resulting from bond formation. Furthermore, large negative activation entropy values indicate strong ordering in the transition state, which is characteristic of single-step cycloaddition reactions. The activation Gibbs free energy difference between the endo- and exo-channels (at 388 K for B97X-D) show that this near-isocaloric barrier relates to experimental isomer distribution. Because the calculated activation free energies for both channels are nearly identical (in Table 4), a Curtin–Hammett flux-weighted average yields an approximately equimolar contribution [50]. Therefore, in Table 3, we report a 50:50 average of the endo and exo KIEs.

**Figure 3:**
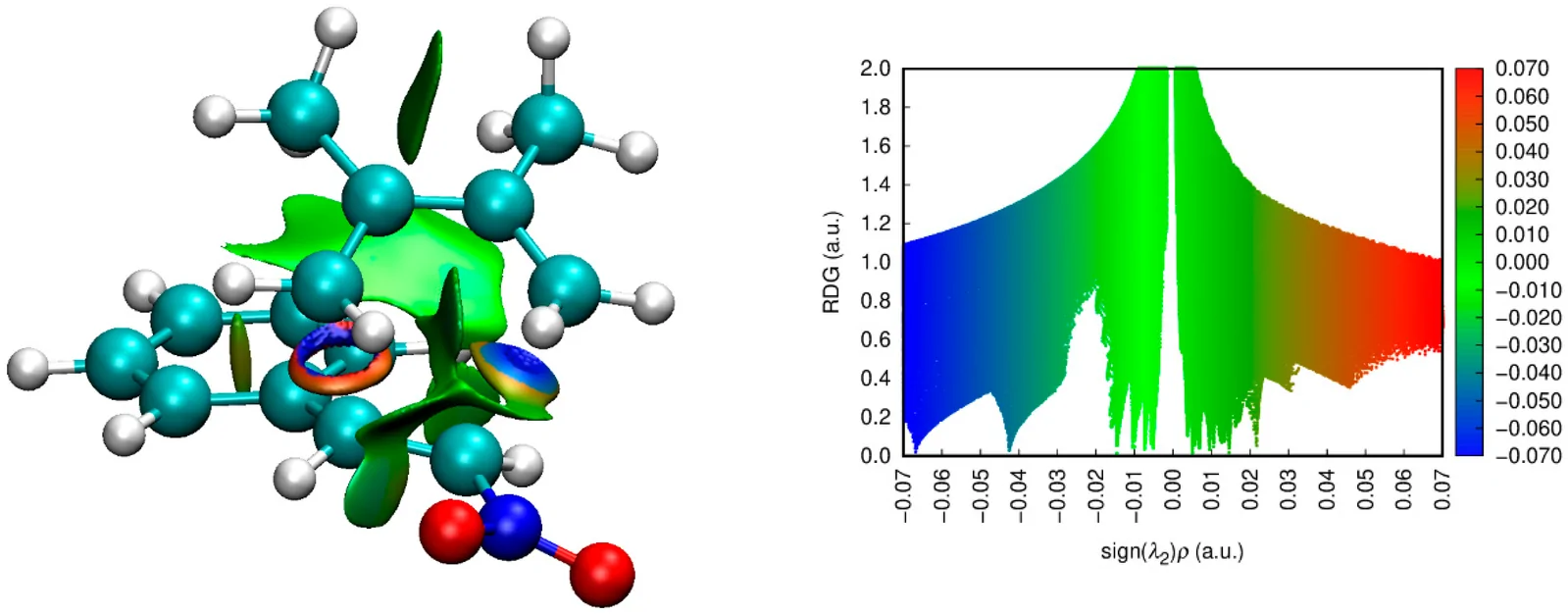
2D and 3D NCI plots for TS exo calculated with ωB97X-D/6-311G(d) level of theory. Color coding is in range −0.07 < sign(λ2)ρ < 0.07 a.u.

**Figure 4:**
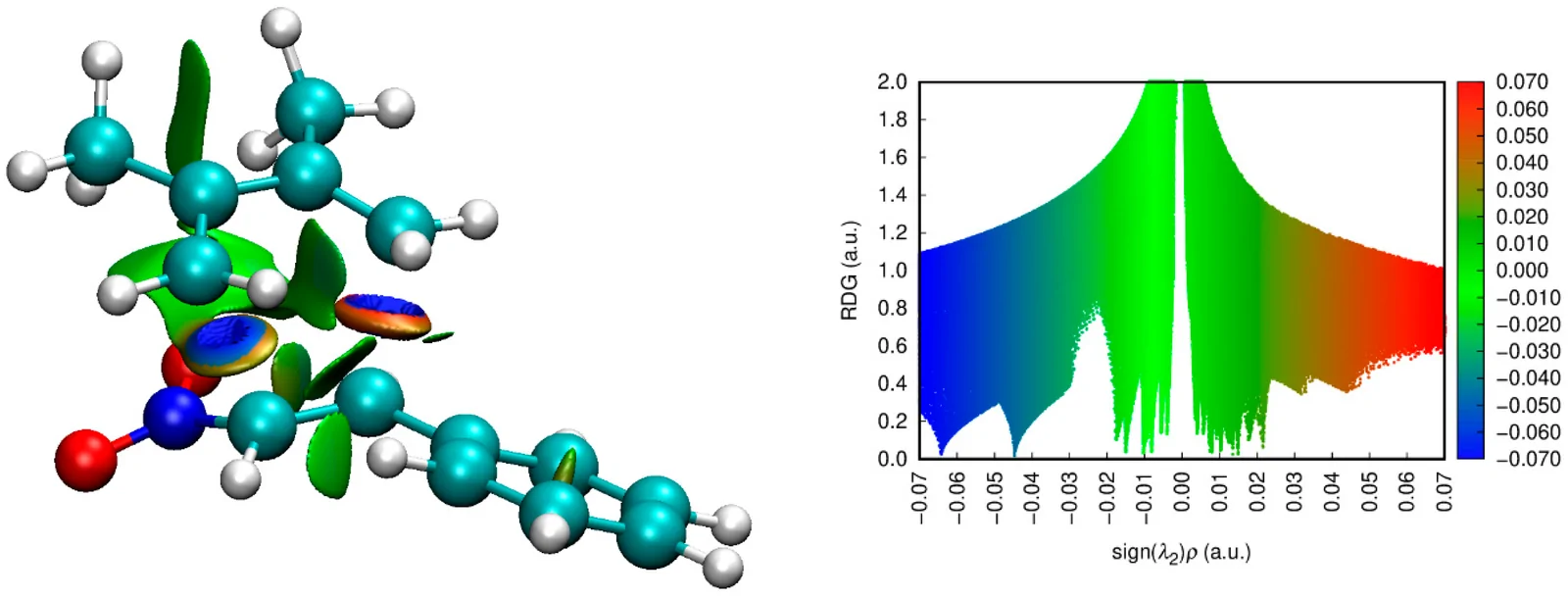
2D and 3D NCI plots for TS endo calculated with ωB97X-D/6-311G(d) level of theory. Color coding is in range −0.07 < sign(λ 2) ρ < 0.07 a.u.

To characterize the forces contributing to the activation energies of two possible reaction paths, we performed an analysis of the non-covalent interactions (NCIs) present in the transition states of the considered reaction. Figure 3 and Figure 4 show a 2D scatter plot of the reduced density gradient function (RDG) versus the product of the electron density ρ and the signum function of the second largest Hessian of ρ, whereas the 3D plot depicts the isosurface of an RDG of 0.5 in the examined structure. In both cases, areas associated with the forming C-C bonds exhibit strong attraction, which is depicted in the form of blue regions (see Figure 3 for TS exo and Figure 4 for TS endo). The transition state of the more favorable *exo* path shows a large area (colored green) of van der Waals interactions of phenyl rings with π bonds of 2,3-dimethylbutadiene (Figure 3), whereas the *endo*-oriented structure possesses a relatively smaller stabilizing region between a nitro group and the same diene fragment. This difference in interaction space size could account for the lower activation barrier, but it is important to remember that NCIs allow for only qualitative visual inspection and do not indicate any quantitative relations.

**Scheme 2:**
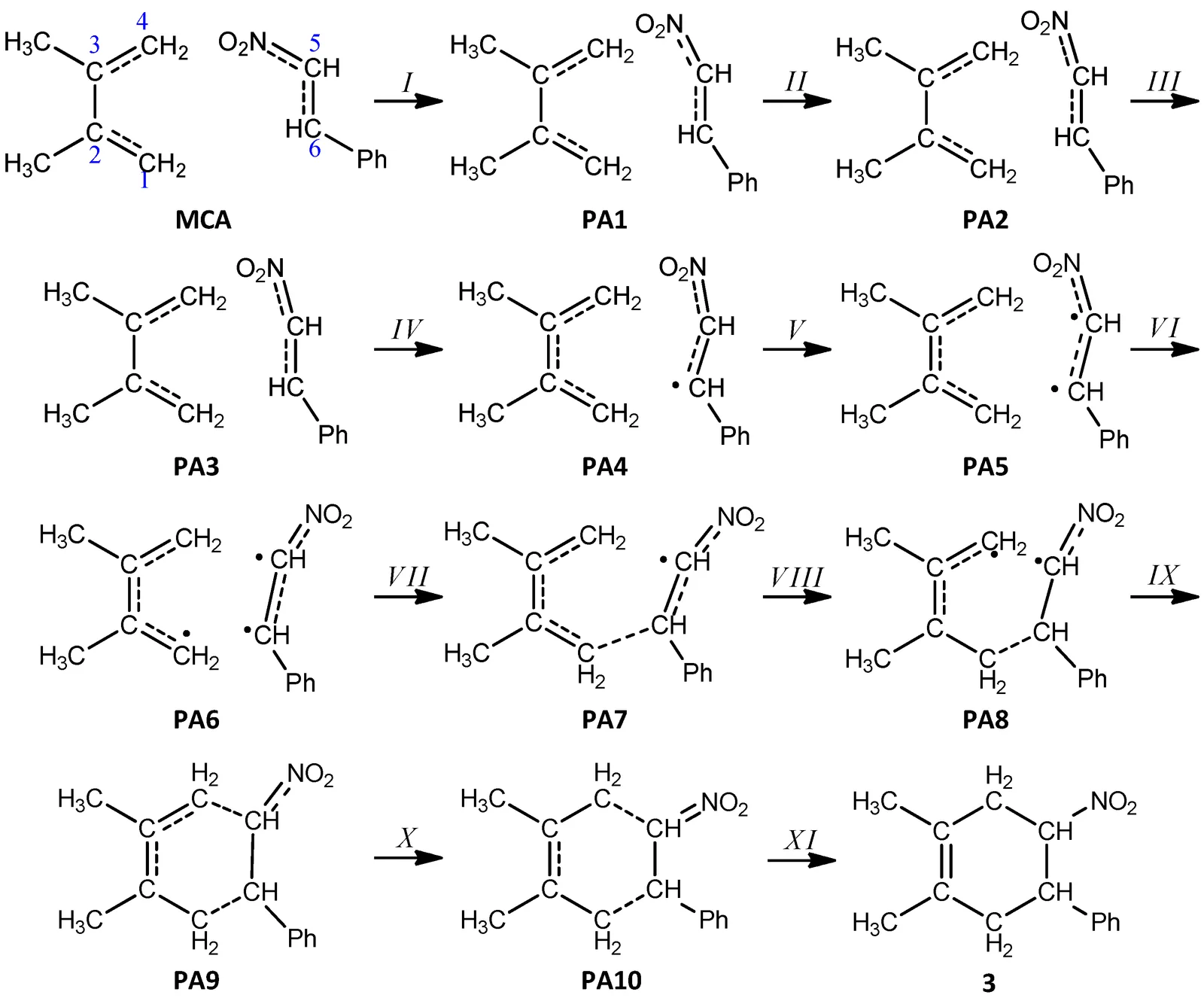
Simplified representation of molecular mechanism of cycloaddition of **1** and **2** via Lewis-like structures based on topological analysis of ELF along reaction path leading to product **3**.

**Scheme 3:**
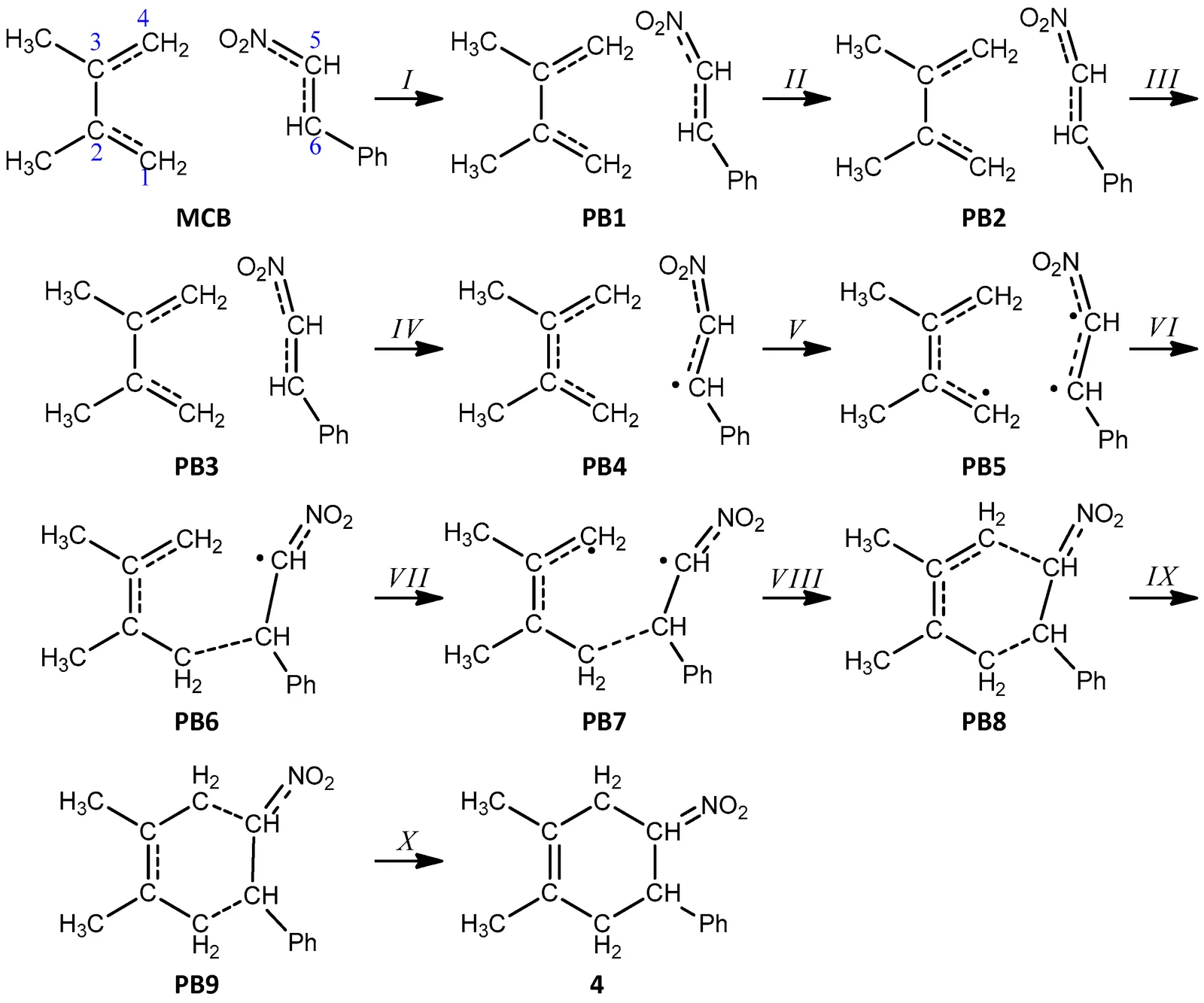
Simplified representation of molecular mechanism of cycloaddition of **1** and **2** via Lewis-like structures based on topological analysis of ELF along reaction path leading to product **4**.

At the end of our studies, to better understand the process of electron density redistribution along the entire reaction path and how new chemical bonds form, we performed an analysis of the electronic structures obtained from the electron localization function (ELF) [51]. The choice of critical points was based on changes in the electronic structure in comparison to the previous one, such as the formation or vanishing of a valence basin. A new phase begins with a new critical point. In Table 4 and Table 5, information on ELF valence basin populations and other data is presented, and Scheme 2 and Scheme 3 present the mechanisms via Lewis-like structures.

**Figure 5:**
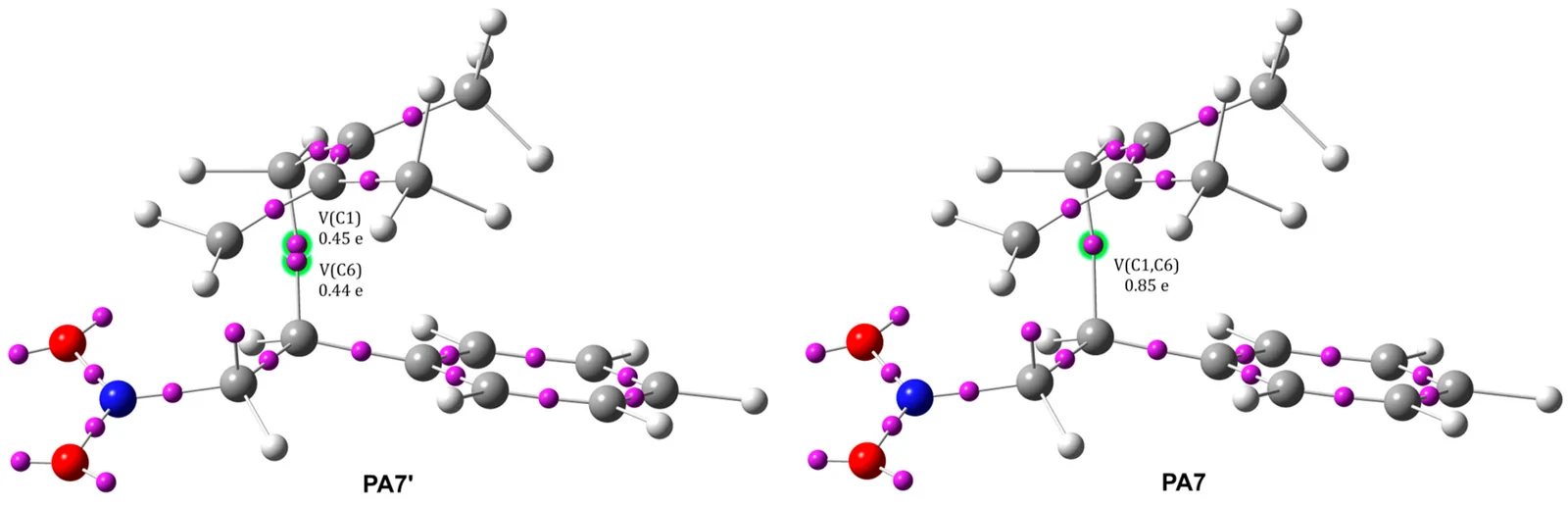

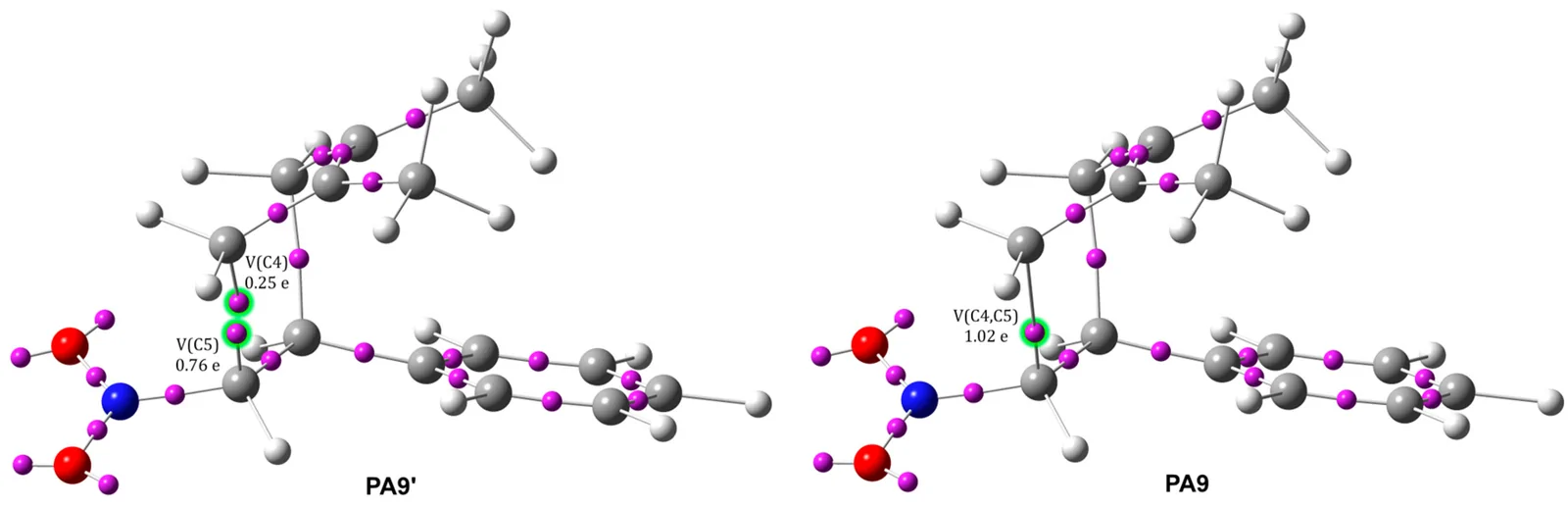
ELF valence basin attractors of structures of most important topological changes and creation of C1-C6 and C4-C5 bonds, during cycloaddition of **1** and **2** along reaction path leading to product **3**. Structures with apostrophe represent first structure before critical point.

We began our study with an analysis of the exo-oriented cycloaddition path, which revealed eleven phases (Scheme 2, Table 5). From molecular complex **MCA** to point **PA6**, the electronic structure of the reagents is mainly rearranged, leading to the creation of *pseudoradical* centers, starting with the carbon (C6) of E-2-phenyl-1-nitroethene **2** in the form of monosynaptic basin V(C6) of 0.14 e. This change takes place at **PA4** with an increase in the energy of the system to 10.1 kcal/mol, just before **TSexo**, and is in full agreement with the results regarding local reactivity (Figure 1). At the next point, **PA5**, a new monosynaptic basin is created, V(C5), integrating 0.40 e, followed by another one, V(C1) of 0.28 e. These changes lead to the formation of the first new bond C1-C6, at d(C1-C6) = 1.989 Å, which occurs by the merging of two *pseudoradical* centers V(C1) of 0.45 e and V(C6) of 0.44 e (see Figure 5, points **PA7′** and **PA7**). The disynaptic basin V(C1,C6) starts with 0.85 e, which represents 46% of the final electron population of that bond. At that time, GEDT amounts to 0.23 e, and the energy of the system is lowered to 7.8 kcal/mol. At the following two points, **PA8** and **PA9**, an additional monosynaptic basin V(C4) with a population of 0.17 e emerges, which soon joins with V(C5), creating the second new bond, d(C4-C5) = 1.886 Å, as shown in Figure 5, points **PA9′** and **PA9**. This change in electronic structure results in a further drop in energy to 1.7 kcal/mol. At the last point, **PA10**, the splitting of the disynaptic basin V(C2,C3) of 3.13 e occurs. In the final steps of the reaction, only alterations in geometry, leading to the energetic minima of the cycloadduct **3**, cause the system’s energy to settle at −48.9 kcal/mol. The *exo*-channel of the Diels–Alder reaction of nitroalkene **2** and butadiene **1** is the result of a *two-stage*, *one-step* mechanism.

**Figure 6:**
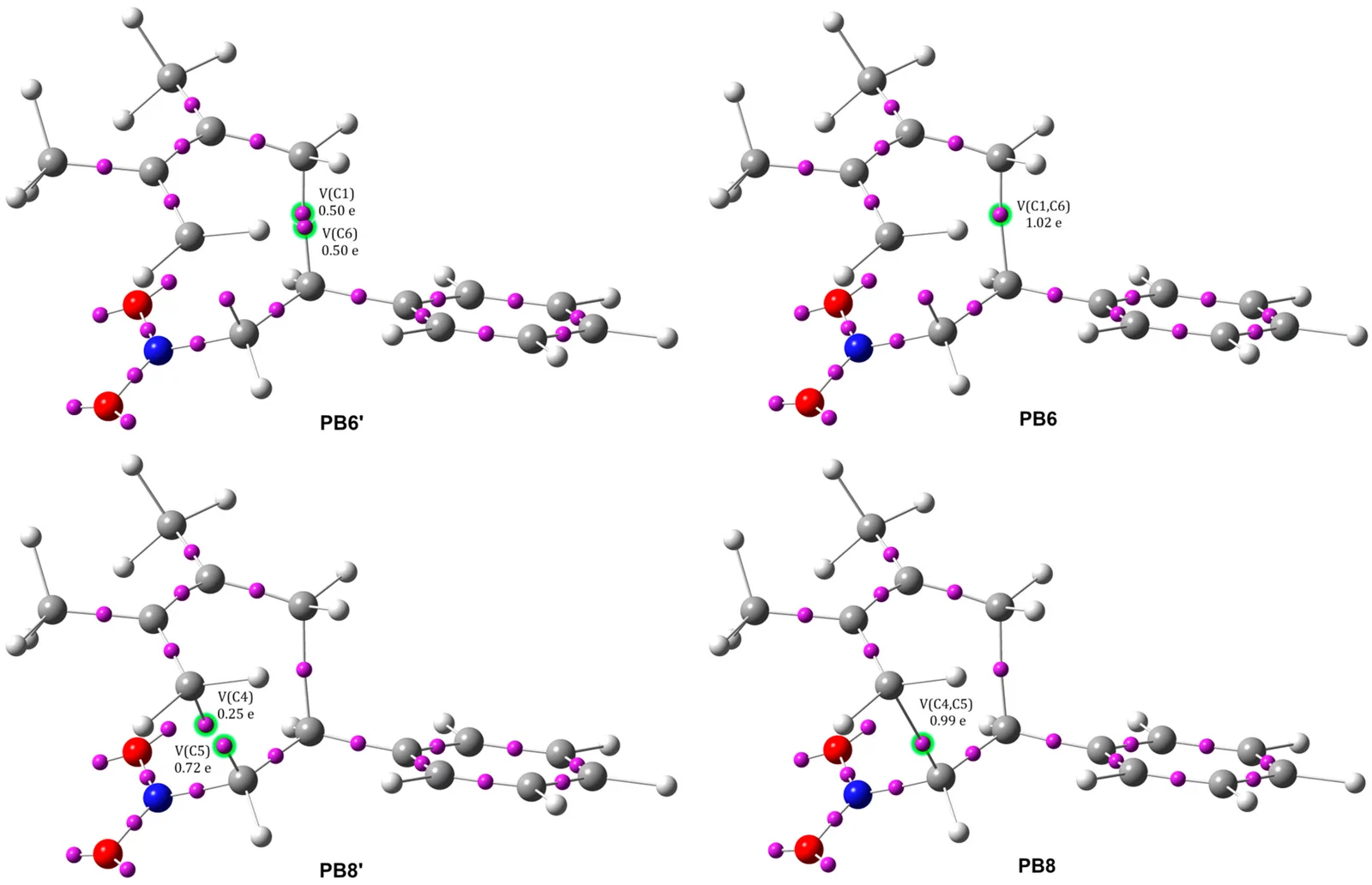
ELF valence basin attractors of structures of most important topological changes and creation of C1-C6 and C4-C5 bonds, during cycloaddition of **1** and **2** along reaction path leading to product **4.** Structures with apostrophe represent first structure before critical point.

Next, we analyzed the reaction path leading to the *endo* isomer of cyclohexene **4**, which involved ten phases (Scheme 3, Table 6). Compared with the *exo* route, all, except one, critical points show analogous electron populations and valence basins with less than 10% deviation in values. In terms of the energy profile and bond lengths, more dynamic changes are visible—a faster rise before the transition state **TSendo** and a drop afterwards coinciding with a slightly quicker advancement in the reduction in the distance between the atoms of developing bonds (Table 5). The main difference in the mechanism resides in the creation of monosynaptic basin V(C1) of 0.27 e simultaneously with V(C5) of 0.36 e at point **PB5**, whereas in the *exo* path, V(C5) appears before V(C1) (points **PA5** and **PA6**). The first new bond is made by the merging of two *pseudoradical* centers at C1 and C6, d(C1-C6) = 2.218 Å, starting phase *VI*, as shown in Figure 6 (points **PB6′** and **PB6**). The system’s energy is 6.7 kcal/mol, and the GEDT is 0.24 e. At the next point **PB7**, monosynaptic basin V(C4) appears with a population of 0.20 e, which soon combines with basin V(C5), producing the second new bond, d(C4,C5) = 1.911 Å (see Figure 6, points **PB8′** and **PB8**). This event marks the beginning of phase *VIII*, showing a drop in energy to 2.7 kcal/mol. As in the *exo* path, the *endo* route proceeds by a *two-stage*, *one-step* mechanism—new bonds are created consecutively, and there is no stable intermediate such as a zwitterion.

## 3. Computational Procedure

All computations were performed using the GAUSSIAN 1675 package on the Helios computer cluster at the regional CYFRONET center in Cracow, Poland. DFT calculations were conducted using different functionals [52]. Geometry optimizations were done using different basis sets [53]. Transition states were located using the QST2 procedure. The applied methodology has been successfully employed for the optimization and characterization of diverse organic cycloaddition components [54], especially for conjugated nitroorganic systems [55,56]. Moreover, the obtained results correlate well with experimental data. All critical structures were computed at T = 388 K and p = 1 atm. Vibrational frequency analysis was performed to characterize stationary points. The harmonic vibrational formalism (e.g., Bigeleisen–Mayer formulation) was used to evaluate isotopic partition function ratios.

Reactants and products were confirmed as minima by the presence of only positive Hessian eigenvalues. All TSs were identified by a single imaginary frequency, associated with one negative Hessian eigenvalue. The Gibbs free energies of the activation and rate constants were calculated using standard thermochemical equations. Kinetic isotope effects were defined as the k_12C_/k_14C_ ratio. Rate constants k were calculated from ΔG values using standard physical equations.

For all optimized TSs, Intrinsic Reaction Coordinate (IRC) calculations were performed to confirm that the located TSs connect the appropriate minimum stationary points associated with the reactants and products. Solvent effects were taken into account using the Self-Consistent Reaction Field (SCRF) [57] within the framework of the polarizable continuum model (PCM) [58]. The presence of toluene was taken into account in the calculations, as this specific solvent was used in [25].

GEDT values were calculated using NPA [59,60]. The GEDT associated with framework (f) was determined as the sum of NPA atomic charges, GEDT(f) = Σqf, where qf represents the charges of the atoms defining framework (f) at the TSs.

The global electronic properties of the reactants were estimated according to the recommendations of Domingo [19,61]. In particular, CDFT indices were calculated in the gas phase and then analyzed according to the electrophilicity and nucleophilicity indices. The electrophilic Pk+ and nucleophilic Pk^−^ Parr functions were derived from changes in the atomic spin densities of reagents [62].

Weak interactions in transition states were calculated using NCIPLOT version 3.0 [63,64] and visualized in VMD 2.0.0 software [65].

Electron localization function (ELF) topological analysis was performed using the TopMod 09 package [51]. The structures used in the analysis were obtained by IRC calculations with a step size of 1; the *exo* path consisted of 765 points, and the *endo* path consisted of 835. Key geometries are collected in the Supplementary Materials.

## 4. Conclusions

Quantum-chemical studies provide deep insight into the stereochemistry and molecular mechanism of the Diels–Alder reaction between 2,3-dimethylbutadiene and (*E*)-2-phenyl-1-nitroethene, a process that can be considered a model for a broader range of moderately polar cycloadditions, with an asynchronous transition state. In particular, as could be expected based on CDFT indices, the bond at carbon atom 2 of the nitrovinyl fragment always forms more rapidly. By utilizing experimentally measured kinetic isotope effects, we were able to precisely evaluate a series of commonly used levels of theory regarding the quantitative description of transition state geometries. It was found that a number of DFT methods handle this type of problem satisfactorily, with the best approximations obtained using the ωB97X-D/6-311G(d) level of theory. Based on the CDFT results, we classified the process—as expected—as moderately polar; this classification was confirmed by the estimates of electron transfer within the localized and verified transition states. Consequently, these findings may prove useful to researchers seeking an optimal computational approach for accurately describing the transition state geometries of cycloadditions with the participation of carbodienes and conjugated nitroalkenes.

NCI analysis showed qualitatively that the transition state of the more favorable *exo* path exhibits a large stabilizing van der Waals interaction area between the nitrostyrene phenyl ring and the diene π bond, whereas the *endo* path possesses a relatively smaller region involving the nitro group.

BET studies provided a detailed description of the electron density redistribution along the entire reaction path. Topological ELF analysis revealed that both reaction paths proceed by a *one-step*, *two-stage* mechanism and that new bonds are created by the merging of two *pseudoradical* centers on carbon atoms.

## Figures and Tables

**Table 1:** Global electronic properties of 2,3-dimethylbutadiene **1** and E-2-phenyl-1-nitroethene **2**.

Component	E_HOMO_ [a.u.]	E_LUMO_ [a.u.]	μ [eV]	η [eV]	ω [eV]	N [eV]
1	−0.3117	0.0616	−3.40	10.16	0.57	2.92
2	−0.3341	−0.0342	−5.01	8.16	1.54	2.31

**Table 2:** Key parameters of transition states of Diels–Alder reaction between 2,3-dimethylbutadiene **1** and E-2-phenyl-1-nitroethene **2** in light of DFT calculations.

Theory Level	*endo* Approach			*exo* Approach		
	Interatomic Distances r [Å]		GEDT [e]	Interatomic Distances r [Å]		GEDT [e]
	C4-C5	C6-C1		C4-C5	C6-C1	
B3LYP/ 6-31G(d)	2.367	2.133	0.18	2.425	2.093	0.18
B3LYP/ 6-31+G(d)	2.525	2.033	0.24	2.537	2.026	0.32
B3LYP/ 6-311G(d)	2.329	2.141	0.18	2.389	2.101	0.18
B3LYP/ 6-311+G(d)	2.439	2.070	0.18	2.475	2.049	0.19
ωB97X-D/ 6-31G(d)	2.387	2.127	0.18	2.406	2.112	0.18
ωB97X-D/ 6-31+G(d)	2.505	2.059	0.26	2.485	2.064	0.35
ωB97X-D/ 6-311G(d)	2.347	2.136	0.18	2.376	2.116	0.18
ωB97X-D/ 6-311+G(d)	2.430	2.086	0.20	2.436	2.080	0.22
M062x/ 6-31G(d)	2.289	2.183	0.18	2.322	2.166	0.18
M062x/ 6-31+G(d)	2.317	2.159	0.34	2.349	2.143	0.44
M062x/ 6-311G(d)	2.274	2.185	0.19	2.308	2.168	0.19
M062x/ 6-311+G(d)	2.300	2.166	0.16	2.333	2.150	0.28

**Table 3:** Experimental and DFT-calculated ^14^C KIE for transition states of Diels–Alder reaction between 2,3-dimethylbutadiene **1** and E-2-phenyl-1-nitroethene **2** (numbering of reaction centers 5 and 6 according to designations in Figure 2).

Theory Level	^14^C KIE on Reaction Sites					
	*endo* Approach		*exo* Approach		Average Value	
	5	6	5	6	5	6
B3LYP/ 6-31G(d)	1.0279	1.0501	1.0252	1.0510	1.0266	1.0506
B3LYP/ 6-31+G(d)	1.0216	1.0595	1.0216	1.0576	1.0216	1.0585
B3LYP/ 6-311G(d)	1.0289	1.0492	1.0261	1.0510	1.0275	1.0501
B3LYP/ 6-311+G(d)	1.0234	1.0557	1.0225	1.0548	1.0229	1.0552
ωB97X-D/ 6-31G(d)	1.0270	1.0492	1.0261	1.0482	1.0266	1.0487
ωB97X-D/ 6-31+G(d)	1.0197	1.0557	1.0225	1.0520	1.0211	1.0538
ωB97X-D/ 6-311G(d)	1.0270	1.0482	1.0252	1.0464	1.0261	1.0473
ωB97X-D/ 6-311+G(d)	1.0216	1.0520	1.0225	1.0501	1.0220	1.0510
M062x/ 6-31G(d)	1.0307	1.0436	1.0298	1.0417	1.0302	1.0427
M062x/ 6-31+G(d)	1.0289	1.0436	1.0289	1.0427	1.0289	1.0431
M062x/ 6-311G(d)	1.0307	1.0408	1.0289	1.0399	1.0298	1.0403
M062x/ 6-311+G(d)	1.0298	1.0436	1.0289	1.0417	1.0293	1.0427
Exp. [25]	-	-	-	-	1.0438	1.0474

**Table 4:** DFT-calculated activation parameters for Diels–Alder reaction between 2,3-dimethylbutadiene **1** and E-2-phenyl-1-nitroethene **2**.

Theory Level	*endo* Approach			*exo* Approach		
	ΔH [kcal/ mol]	ΔS [cal/ molK]	ΔG [kcal/ mol]	ΔH [kcal/ mol]	ΔS [cal/ molK]	ΔG [kcal/ mol]
B3LYP/ 6-31G(d)	19.2	−47.9	37.8	19.7	−47.6	38.2
B3LYP/ 6-31+G(d)	21.2	−46.8	39.3	21.7	−47.3	40.1
B3LYP/ 6-311G(d)	20.7	−47.9	39.3	21.5	−47.8	40.0
B3LYP/ 6-311+G(d)	21.5	−47.9	40.0	21.9	−47.6	40.4
ωB97X-D/ 6-31G(d)	10.9	−49.3	30.0	10.4	−51.0	30.2
ωB97X-D/ 6-31+G(d)	12.3	−48.9	31.2	11.9	−50.6	31.5
ωB97X-D/ 6-311G(d)	11.6	−49.3	30.7	11.2	−50.8	30.9
ωB97X-D/ 6-311+G(d)	12.2	−49.0	31.2	11.6	−51.2	31.5
M062x/ 6-31G(d)	8.5	−49.2	27.6	8.7	−51.5	28.7
M062x/ 6-31+G(d)	10.2	−50.0	29.6	10.3	−51.0	30.1
M062x/ 6-311G(d)	8.3	−49.7	27.6	8.5	−51.8	28.6
M062x/ 6-311+G(d)	9.2	−50.4	28.8	9.0	−51.8	29.1

**Table 5:** ELF valence basin populations of IRC points, molecular complex MCA—product **3**—defining eleven different phases characterizing exo-oriented cycloaddition of **1** and **2**. Stationary point TSexo is also included. Distances are given in angstroms, Å; electron populations in average number of electrons, [e]; relative energies in kcal∙mol^−1^; and GEDT values in average number of electrons, [e].

Structures	MCA	PA1	PA2	PA3	PA4	TSexo	PA5	PA6	PA7	PA8	PA9	PA10	3
Phases	I	II	III	IV	V		VI	VII	VIII	IX	X	XI	
*d*1(C1-C6)	3.408	2.661	2.565	2.498	2.396	2.376	2.358	2.351	2.278	2.250	2.189	2.094	1.550
*d*2(C4-C5)	3.380	2.502	2.376	2.286	2.143	2.116	2.093	2.084	1.989	1.954	1.886	1.794	1.529
GEDT	0.01	0.08	0.11	0.14	0.19	0.20	0.21	0.21	0.23	0.24	0.23	0.21	0.08
*d*E	−8.0	1.6	5.0	7.5	10.1	10.2	10.1	10.1	7.8	6.1	1.7	−6.7	−48.9
V(C1,C2)	1.75	1.73	3.23	3.19	3.10	3.08	3.07	2.78	2.55	2.47	2.35	2.23	2.02
V′(C1,C2)	1.68	1.56											
V(C1,C6)									0.85	1.02	1.21	1.41	1.85
V(C1)								0.28					
V(C2,C3)	2.19	2.30	2.35	2.41	2.58	2.63	2.67	2.68	2.89	2.97	3.13	1.74	1.85
V′(C2,C3)												1.56	1.78
V(C3,C4)	1.77	1.70	1.70	3.21	3.10	3.07	3.04	3.03	2.92	2.71	2.53	2.36	1.99
V′(C3,C4)	1.67	1.62	1.57										
V(C4,C5)											1.02	1.23	1.89
V(C4)										0.17			
V(C5,C6)	1.75	3.40	3.37	3.35	3.25	3.22	2.80	2.78	2.53	2.43	2.31	2.19	1.94
V′(C5,C6)	1.73												
V(C5)							0.40	0.41	0.60	0.66			
V(C6)					0.14	0.19	0.24	0.26					

**Table 6:** ELF valence basin populations of IRC points, molecular complex MCB—product **4**—defining ten different phases characterizing endo-oriented cycloaddition of **1** and **2**. Stationary point TSendo is also included. Distances are given in angstroms, Å; electron populations in average number of electrons, [e]; relative energies in kcal∙mol^−1^; and GEDT values in average number of electrons, [e].

Structures	MCB	PB1	PB2	PB3	PB4	TSendo	PB5	PB6	PB7	PB8	PB9	4
Phases	I	II	III	IV	V		VI	VII	VIII	IX	X	
*d*1(C1-C6)	3.239	2.604	2.527	2.495	2.353	2.347	2.325	2.218	2.210	2.163	2.077	1.538
*d*2(C4-C5)	3.556	2.475	2.376	2.335	2.145	2.136	2.108	1.973	1.964	1.911	1.828	1.543
GEDT	0.00	0.09	0.12	0.13	0.20	0.21	0.21	0.24	0.24	0.23	0.22	0.08
*d*E	−7.5	3.4	6.1	7.3	10.7	10.7	10.6	6.7	6.2	2.7	−4.8	−48.2
V(C1,C2)	1.76	1.72	3.23	3.20	3.10	3.08	2.81	2.49	2.47	2.38	2.26	2.02
V′(C1,C2)	1.68	1.57										
V(C1,C6)								1.02	1.05	1.19	1.38	1.83
V(C1)							0.27					
V(C2,C3)	2.19	2.31	2.36	2.38	2.60	2.61	2.66	2.95	2.98	3.10	1.71	1.79
V′(C2,C3)											1.55	1.84
V(C3,C4)	1.79	1.74	1.51	3.24	3.09	3.09	3.05	2.89	2.67	2.54	2.38	2.02
V′(C3,C4)	1.65	1.57	1.77									
V(C4,C5)										0.99	1.19	1.88
V(C4)									0.20			
V(C5,C6)	1.75	3.39	3.38	3.37	3.22	3.22	2.81	2.46	2.44	2.33	2.21	1.97
V′(C5,C6)	1.73											
V(C5)							0.36	0.62	0.64			
V(C6)					0.18	0.18	0.25					

## Data Availability

The original contributions presented in this study are included in the article. Further inquiries can be directed to the corresponding author(s).

## Supplementary Materials

The following supporting information can be downloaded at: https://www.mdpi.com/article/10.3390/ijms27209223/s1.

## References

[1] Diels O., Alder K. (1928). Synthesen in Der Hydroaromatischen Reihe. Justus Liebigs Ann. Chem..

[2] Sadowski M., Kula K. (2024). Nitro-Functionalized Analogues of 1,3-Butadiene: An Overview of Characteristic, Synthesis, Chemical Transformations and Biological Activity. Curr. Chem. Lett..

[3] Kula K. (2026). Recent Progress in the Application of Five-Membered Heterocyclic Analogues of the Nitrobutadiene in Cycloaddition Reactions (Microreview). Chem. Heterocycl. Comp..

[4] Nicolaou K.C., Snyder S.A., Montagnon T., Vassilikogiannakis G. (2002). The Diels-Alder Reaction in Total Synthesis. Angew. Chem. Int. Ed..

[5] Juhl M., Tanner D. (2009). Recent Applications of Intramolecular Diels–Alder Reactions to Natural Product Synthesis. Chem. Soc. Rev..

[6] Houk K.N., Lin Y.T., Brown F.K. (1986). Evidence for the Concerted Mechanism of the Diels-Alder Reaction of Butadiene with Ethylene. J. Am. Chem. Soc..

[7] Jasiński R., Dresler E. (2020). On the Question of Zwitterionic Intermediates in the [3+2] Cycloaddition Reactions: A Critical Review. Organics.

[8] Jasiński R. (2021). On the Question of Stepwise [4+2] Cycloaddition Reactions and Their Stereochemical Aspects. Symmetry.

[9] Kącka-Zych A. (2025). Synthesis of Heterocycles via Cycloaddition Reactions with the Participation of Cyano-Substituted Nitroalkenes (Microreview). Chem. Heterocycl. Comp..

[10] Sadowski M., Dresler E., Wróblewska A., Jasiński R. (2024). A New Insight into the Molecular Mechanism of the Reaction between 2-Methoxyfuran and Ethyl (Z)-3-Phenyl-2-Nitroprop-2-Enoate: An Molecular Electron Density Theory (MEDT) Computational Study. Molecules.

[11] Kula K., Kącka-Zych A., Łapczuk-Krygier A., Jasiński R. (2021). Analysis of the Possibility and Molecular Mechanism of Carbon Dioxide Consumption in the Diels-Alder Processes. Pure Appl. Chem..

[12] Domingo L.R. (2025). Performing a Relative Interacting Atomic Energy Analysis of the Diels-Alder Reaction between Butadiene and Ethylene. Sci. Radices.

[13] Eyring H. (1935). The Activated Complex in Chemical Reactions. J. Chem. Phys..

[14] Jasiński R., Mróz K. (2015). Kinetic Aspects of [3+2] Cycloaddition Reactions between (E)-3,3,3-Trichloro-1-Nitroprop-1-Ene and Ketonitrones. Reac. Kinet. Mech. Cat..

[15] Demchuk O.M., Jasiński R., Pietrusiewicz K.M. (2015). New Insights into the Mechanism of Reduction of Tertiary Phosphine Oxides by Means of Phenylsilane. Heteroat. Chem..

[16] Hammett L.P. (1937). The Effect of Structure upon the Reactions of Organic Compounds. Benzene Derivatives. J. Am. Chem. Soc..

[17] Hammett L.P. (1935). Some Relations between Reaction Rates and Equilibrium Constants. Chem. Rev..

[18] Jasiński R., Kubik M., Łapczuk-Krygier A., Kącka A., Dresler E., Boguszewska-Czubara A. (2014). An Experimental and Theoretical Study of the Hetero Diels–Alder Reactions between (E)-2-Aryl-1-Cyano-1-Nitroethenes and Ethyl Vinyl Ether: One-Step or Zwitterionic, Two-Step Mechanism?. Reac. Kinet. Mech. Cat..

[19] Domingo L.R., Ríos-Gutiérrez M. (2023). A Useful Classification of Organic Reactions Based on the Flux of the Electron Density. Sci. Radices.

[20] Kula K., Łapczuk A., Woliński P., Sadowski M. (2025). On the Question of the Correlation between Kinetic Dimroth Parameters and Global Electron Density Transfer in [4+2]-π-Electron Cycloaddition Reactions. Curr. Chem. Lett..

[21] Dutkiewicz M., Dutkiewicz E. (1993). The Relation between the Nonlinear Dielectric Effect and Solvent Polarity. J. Solut. Chem..

[22] Langhals H. (2023). How the Concept of Solvent Polarity Investigated with Solvatochromic Probes Helps Studying Intermolecular Interactions. Liquids.

[23] Mao Z., Campbell C.T. (2020). Kinetic Isotope Effects: Interpretation and Prediction Using Degrees of Rate Control. ACS Catal..

[24] Karandashev K., Xu Z.-H., Meuwly M., Vaníček J., Richardson J.O. (2017). Kinetic Isotope Effects and How to Describe Them. Struct. Dyn..

[25] Kupczyk-Subotkowska L., Shine H.J. (1993). Carbon-14-KIE in the Diels-Alder Addition of .Beta.-Nitrostyrene to 2,3-Dimethylbutadiene. Addition Is Deduced to Be Concerted. J. Am. Chem. Soc..

[26] Domingo L., Ríos-Gutiérrez M., Pérez P. (2016). Applications of the Conceptual Density Functional Theory Indices to Organic Chemistry Reactivity. Molecules.

[27] Sadowski M., Utnicka J., Wójtowicz A., Kula K. (2023). The Global and Local Reactivity of C,N-Diarylnitryle Imines in [3+2] Cycloaddition Processes with Trans-β-Nitrostyrene According to Molecular Electron Density Theory: A Computational Study. Curr. Chem. Lett..

[28] Kula K., Sadowski M. (2023). Regio- and Stereoselectivity of [3+2] Cycloaddition Reactions between (Z)-1-(Anthracen-9-Yl)-N-Methyl Nitrone and Analogs of Trans-β-Nitrostyrene on the Basis of MEDT Computational Study. Chem. Heterocycl. Comp..

[29] Woliński P., Zawadzińska-Wrochniak K., Dresler E., Jasiński R. (2025). On the Question of the Course of the Hetero Diels–Alder Reactions Between N-(2,2,2-Trichloroethylidene)Carboxamides and Dicyclohexylcarbodiimide: A New Case of the Stepwise Zwitterionic Cycloaddition Process. Molecules.

[30] Woliński P., Dresler E., Jasiński R. (2025). A New Mechanistic Insight into the Molecular Mechanisms of the Addition Reactions of 2-Aryl-3-Nitro-2 *H* -Chromenes to Pyrazoles and Cyclopentadienes. New J. Chem..

[31] Domingo L.R., Aurell M.J., Pérez P., Contreras R. (2002). Quantitative Characterization of the Global Electrophilicity Power of Common Diene/Dienophile Pairs in Diels–Alder Reactions. Tetrahedron.

[32] Domingo L.R., Sáez J.A. (2009). Understanding the Mechanism of Polar Diels–Alder Reactions. Org. Biomol. Chem..

[33] Dresler E., Wróblewska A., Jasiński R. (2022). Understanding the Regioselectivity and the Molecular Mechanism of [3 + 2] Cycloaddition Reactions between Nitrous Oxide and Conjugated Nitroalkenes: A DFT Computational Study. Molecules.

[34] Sadowski M., Dresler E., Zawadzińska K., Wróblewska A., Jasiński R. (2024). Syn-Propanethial S-Oxide as an Available Natural Building Block for the Preparation of Nitro-Functionalized, Sulfur-Containing Five-Membered Heterocycles: An MEDT Study. Molecules.

[35] Zawadzińska K., Kula K. (2021). Application of β-Phosphorylated Nitroethenes in [3+2] Cycloaddition Reactions Involving Benzonitrile N-Oxide in the Light of a DFT Computational Study. Organics.

[36] Zawadzińska-Wrochniak K., Gaurav G.K., Demchuk O.M. (2025). Synthesis and Physical Properties of the (E)-3,3,3-Tribromo-1-Nitroprop-1-Ene. Sci. Radices.

[37] Sadowski M., Kula K. (2024). Unexpected Course of Reaction Between (1E,3E)-1,4-Dinitro-1,3-Butadiene and N-Methyl Azomethine Ylide—A Comprehensive Experimental and Quantum-Chemical Study. Molecules.

[38] Kula K., Jasiński R. (2024). Synthesis of Bis(Het)Aryl Systems via Domino Reaction Involving (2E,4E)-2,5-Dinitrohexa-2,4-Diene: DFT Mechanistic Considerations. Chem. Heterocycl. Comp..

[39] Kula K., Jasiński R. (2026). A New, Comprehensive Insight into the Competition between [4+2] and [2+4] Cycloaddition Schemes and Molecular Mechanism of the Diels–Alder Reaction of 2-Nitrofuran with Isoprene. New J. Chem..

[40] Bigosińska A., Gondek D., Domingo L.R., Kącka-Zych A. (2026). Electrophilic Allenes Participate in Polar Diels–Alder Reactions: Study of the Reactivity and Site-, Regio- and Stereoselectivity from a Molecular Electron Density Theory Perspective. RSC Adv..

[41] Kącka-Zych A., Zeroual A., Syed A., Bahkali A.H. (2025). Docking Survey, ADME, Toxicological Insights, and Mechanistic Exploration of the Diels–Alder Reaction Between Hexachlorocyclopentadiene and Dichloroethylene. J. Comput. Chem..

[42] Jasiński R. (2014). Searching for Zwitterionic Intermediates in Hetero Diels–Alder Reactions between Methyl α,p-Dinitrocinnamate and Vinyl-Alkyl Ethers. Comput. Theor. Chem..

[43] Kalla E., Semrád H., Maier L., Paruch K., Zipse H., Munzarová M. (2025). Exploring the Regioselectivity of Diels–Alder Reactions of 2,6-Disubstituted Benzoquinones through DFT Calculations. Eur. J. Org. Chem..

[44] Ameur S., Barhoumi A., Abdallaoui H.E.A.E., Syed A., Belghiti M.E., Elgorban A.M., Wong L.S., Wang S., Idrissi M.E., Zeroual A. (2024). Molecular Docking, Exploring Diverse Selectivities and Mechanistic Insights in the Cycloaddition Reaction between 3-Benzoylpyrrolo-[1,2-a]Quinoxaline-1,2,4(5H)-Triones and Butyl Vinyl Ether. Chem. Heterocycl. Comp..

[45] Horváth Á., Benkő Z. (2022). Understanding the Mechanism of Diels–Alder Reactions with Anionic Dienophiles: A Systematic Comparison of [ECX]^−^ (E = P, As; X = O, S, Se) Anions. Inorg. Chem..

[46] Kostiv I., Marshalok O., Marshalok G., Pyrig I. (2015). Effect of the Reactants Molar Ratio on the Kinetics of the Reaction to Obtain 2-Hydroxyethyl-1,3,4 Trimethyl-Cyclohex-3-Encarboxylate. Chem. Chem. Techmol..

[47] Polevaya I.S., Makitra G.G., Marshalok G.A., Kovalskyi Y.P. (2012). Effect of the Reactants Molar Ratio on the Kinetics of Cycloaddition of 2,3-Dimethylbuta-1,3-Diene to Allyl Methacrylate. Russ. J. Gen. Chem..

[48] Szczepanek A., Jasińska E., Kącka A., Jasiński R. (2015). An Experimental and Quantumchemical Study of [2+3] Cycloaddition between (Z)-C-(m,m,p-Trimethoxyphenyl)-N-(p-Methyphenyl)-Nitrone and (E)-3,3,3-Trichloro-1-Nitroprop-1-Ene: Mechanistic Aspects. Curr. Chem. Lett..

[49] Jasiński R. (2009). Regio- and Stereoselectivity of [2+3]Cycloaddition of Nitroethene to (Z)-N-Aryl-C-Phenylnitrones. Collect. Czech. Chem. Commun..

[50] Seeman J.I. (1983). Effect of Conformational Change on Reactivity in Organic Chemistry. Evaluations, Applications, and Extensions of Curtin-Hammett Winstein-Holness Kinetics. Chem. Rev..

[51] Noury S., Krokidis X., Fuster F., Silvi B. (1997). TopMod Package. https://wiki.ch.ic.ac.uk/images/3/3e/Manual.pdf.

[52] Chai J.-D., Head-Gordon M. (2008). Long-Range Corrected Hybrid Density Functionals with Damped Atom–Atom Dispersion Corrections. Phys. Chem. Chem. Phys..

[53] Krishnan R., Binkley J.S., Seeger R., Pople J.A. (1980). Self-Consistent Molecular Orbital Methods. XX. A Basis Set for Correlated Wave Functions. J. Chem. Phys..

[54] Kula K., Jasiński R. (2025). On the Question of the Application Potential and the Molecular Mechanism of the Formation of 1,3-Diaryl-5-Nitropyrazoles from Trichloromethylated Diarylnitropyrazolines. Molecules.

[55] Kula K., Kuś E. (2025). In Silico Study About Substituent Effects, Electronic Properties, and the Biological Potential of 1,3-Butadiene Analogues. Int. J. Mol. Sci..

[56] Sadowski M., Synkiewicz-Musialska B., Kula K. (2024). (1E,3E)-1,4-Dinitro-1,3-Butadiene—Synthesis, Spectral Characteristics and Computational Study Based on MEDT, ADME and PASS Simulation. Molecules.

[57] Tomasi J., Persico M. (1994). Molecular Interactions in Solution: An Overview of Methods Based on Continuous Distributions of the Solvent. Chem. Rev..

[58] Cossi M., Barone V., Cammi R., Tomasi J. (1996). Ab Initio Study of Solvated Molecules: A New Implementation of the Polarizable Continuum Model. Chem. Phys. Lett..

[59] Reed A.E., Weinstock R.B., Weinhold F. (1985). Natural Population Analysis. J. Chem. Phys..

[60] Reed A.E., Curtiss L.A., Weinhold F. (1988). Intermolecular Interactions from a Natural Bond Orbital, Donor-Acceptor Viewpoint. Chem. Rev..

[61] Domingo L. (2016). Molecular Electron Density Theory: A Modern View of Reactivity in Organic Chemistry. Molecules.

[62] Domingo L.R., Pérez P., Sáez J.A. (2013). Understanding the Local Reactivity in Polar Organic Reactions through Electrophilic and Nucleophilic Parr Functions. RSC Adv..

[63] Johnson E.R., Keinan S., Mori-Sánchez P., Contreras-García J., Cohen A.J., Yang W. (2010). Revealing Noncovalent Interactions. J. Am. Chem. Soc..

[64] Contreras-García J., Johnson E.R., Keinan S., Chaudret R., Piquemal J.-P., Beratan D.N., Yang W. (2011). NCIPLOT: A Program for Plotting Noncovalent Interaction Regions. J. Chem. Theory Comput..

[65] Humphrey W., Dalke A., Schulten K. (1996). VMD: Visual Molecular Dynamics. J. Mol. Graph..

