# Supplementary material for: A Comprehensive View on the Molecular Mechanism of the Diels–Alder Reaction Between 2,3-Dimethylbutadiene and E-2-Phenyl-1-Nitroethene: CDFT/ELF, 14C Kinetic Isotope Effects, Electron Density Transfers, Reaction Profiles and BET Analyses
Source: Int J Mol Sci. 2026 Oct 17;27(20):9223. doi: 10.3390/ijms27209223 (PMC13705300; doi:10.3390/ijms27209223)
Supplement: Supplementary file 1 [file ijms-27-09223-s001.zip › ijms-4610327-supplementary.pdf]

Supplementary Material

A Comprehensive View on the Molecular Mechanism of the Diels–Alder Reaction Between 2,3-Dimethylbutadiene and E-2-Phenyl-1-Nitroethene: CDFT/ELF, ^{14}C Kinetic Isotope Effects, Electron Density Transfers, Reaction Profiles and BET Analyses

Przemysław Woliński, Weronika Łącka and Radomir Jasiński *

Cracow University of Technology, Faculty of Chemical Engineering and Technology, Warszawska 24, 31-155 Cracow, Poland; przemyslaw.wolinski@pk.edu.pl (P.W.); weronika.lacka@student.pk.edu.pl (W.Ł.)

* Correspondence: radomir.jasinski@pk.edu.pl

KEY GEOMETRIES:

B3LYP/6-31G(d)

TS endo

Center Number	Atomic Number	Atomic Type	Coordinates (Angstroms) X	Y	Z
1	6	0	-2.377037	0.606036	0.973625
2	6	0	-1.646705	1.606455	0.269035
3	6	0	-1.692130	-0.342145	1.711399
4	1	0	-0.726197	-0.140845	2.157181
5	1	0	-2.222877	-1.192442	2.131197
6	6	0	-0.252829	1.632130	0.349639
7	1	0	0.290784	2.374170	-0.229724
8	1	0	0.246223	1.349725	1.269015
9	6	0	-3.860674	0.437353	0.728853
10	1	0	-4.069373	0.215602	-0.324206
11	1	0	-4.268177	-0.377795	1.332811
12	1	0	-4.410869	1.350917	0.986497
13	6	0	-2.354657	2.486430	-0.733485
14	1	0	-3.137483	3.083050	-0.248024
15	1	0	-1.658414	3.178784	-1.214475
16	1	0	-2.843575	1.893060	-1.515149
17	6	0	-0.336486	-1.240875	-0.007961
18	1	0	0.050586	-2.013355	0.639876
19	6	0	0.434956	-0.186848	-0.527632
20	1	0	0.146450	0.136483	-1.521614
21	6	0	1.880664	-0.081955	-0.217111
22	6	0	2.739971	0.512829	-1.156670
23	6	0	2.433510	-0.543069	0.990899
24	6	0	4.106526	0.629525	-0.906832
25	1	0	2.329123	0.878630	-2.094709
26	6	0	3.799549	-0.424816	1.241723
27	1	0	1.794916	-0.998438	1.743416
28	6	0	4.642971	0.160572	0.294109
29	1	0	4.752529	1.085987	-1.651897
30	1	0	4.206271	-0.790957	2.180541
31	1	0	5.707470	0.251154	0.491122
32	7	0	-1.466241	-1.690805	-0.756602
33	8	0	-1.954843	-0.929578	-1.611204
34	8	0	-1.923842	-2.815151	-0.500446

B3LYP/6-31G(d)

TS exo

Center Number	Atomic Number	Atomic Type	Coordinates (Angstroms) X	Y	Z
1	6	0	0.401943	1.744995	0.987955
2	6	0	1.255192	1.999101	-0.125528
3	6	0	0.585135	0.590760	1.756293
4	1	0	1.575897	0.181000	1.924564
5	1	0	-0.111672	0.404058	2.570200
6	6	0	2.323001	1.160016	-0.373744
7	1	0	2.897610	1.260165	-1.290668
8	1	0	2.817260	0.616437	0.420875
9	6	0	-0.838304	2.581831	1.187368
10	1	0	-1.378698	2.282844	2.088960
11	1	0	-0.584953	3.645270	1.280916
12	1	0	-1.528136	2.492940	0.337561
13	6	0	0.892647	3.075341	-1.127886
14	1	0	-0.123818	2.956414	-1.522417
15	1	0	0.936411	4.070371	-0.666630
16	1	0	1.587425	3.074355	-1.972297
17	6	0	0.205086	-1.001491	0.452457
18	1	0	0.381250	-1.677444	1.284182
19	6	0	1.224153	-0.996705	-0.518362
20	1	0	1.053857	-0.898001	-1.579116
21	6	0	-1.214810	-0.858648	0.051668
22	6	0	-2.213333	-1.431619	0.857613
23	6	0	-1.610218	-0.174352	-1.110897
24	6	0	-3.560189	-1.331133	0.513167
25	1	0	-1.925951	-1.966854	1.759665
26	6	0	-2.957938	-0.072458	-1.455222
27	1	0	-0.862523	0.282328	-1.752819
28	6	0	-3.938978	-0.650331	-0.645989
29	1	0	-4.313779	-1.788359	1.148644
30	1	0	-3.241697	0.457419	-2.360616
31	1	0	-4.988001	-0.572305	-0.917437
32	7	0	2.444261	-1.671910	-0.232491
33	8	0	2.753590	-1.872965	0.959612
34	8	0	3.168832	-1.993173	-1.187048

B3LYP/6-31+G(d)

TS endo

Center Number	Atomic Number	Atomic Type	Coordinates (Angstroms) X	Y	Z
1	6	0	-2.431206	0.623395	0.960788
2	6	0	-1.653221	1.573033	0.227377
3	6	0	-1.799294	-0.296766	1.769128
4	1	0	-0.811006	-0.136489	2.179416
5	1	0	-2.358910	-1.117758	2.209514
6	6	0	-0.251827	1.559957	0.333348
7	1	0	0.305388	2.302806	-0.232842
8	1	0	0.203649	1.324272	1.289233
9	6	0	-3.917724	0.502978	0.701173
10	1	0	-4.124884	0.270382	-0.349823
11	1	0	-4.359925	-0.286019	1.315791
12	1	0	-4.435576	1.441058	0.938358
13	6	0	-2.315653	2.446512	-0.808127
14	1	0	-3.064058	3.101567	-0.342313
15	1	0	-1.586368	3.082238	-1.317849
16	1	0	-2.841607	1.850090	-1.563467
17	6	0	-0.301660	-1.266606	-0.017758
18	1	0	0.084011	-2.009802	0.664018
19	6	0	0.432365	-0.161947	-0.504439
20	1	0	0.163664	0.133340	-1.514280
21	6	0	1.887271	-0.059201	-0.197261
22	6	0	2.750104	0.489606	-1.161216
23	6	0	2.436101	-0.481384	1.026655
24	6	0	4.120848	0.600968	-0.918100
25	1	0	2.342660	0.823696	-2.112925
26	6	0	3.805616	-0.368915	1.271789
27	1	0	1.795020	-0.902704	1.797265
28	6	0	4.654717	0.171681	0.300241
29	1	0	4.770414	1.021117	-1.681641
30	1	0	4.210152	-0.704535	2.223225
31	1	0	5.720881	0.257585	0.492243
32	7	0	-1.426171	-1.715677	-0.741933
33	8	0	-1.932525	-0.953797	-1.594958
34	8	0	-1.883591	-2.851324	-0.492790

B3LYP/6-31+G(d)

TS exo

Center Number	Atomic Number	Atomic Type	Coordinates (Angstroms) X	Y	Z
1	6	0	0.440908	1.731422	0.979615
2	6	0	1.317282	2.007209	-0.115714
3	6	0	0.597361	0.549506	1.723396
4	1	0	1.589453	0.150062	1.914679
5	1	0	-0.102955	0.375563	2.537717
6	6	0	2.391253	1.178938	-0.359569
7	1	0	2.980141	1.290884	-1.266168
8	1	0	2.845513	0.578904	0.417925
9	6	0	-0.787403	2.581556	1.186273
10	1	0	-1.345799	2.266916	2.071545
11	1	0	-0.515460	3.637434	1.312676
12	1	0	-1.466571	2.531388	0.324129
13	6	0	0.979887	3.114014	-1.094653
14	1	0	-0.027262	3.007381	-1.516158
15	1	0	1.015460	4.094951	-0.603392
16	1	0	1.694543	3.134740	-1.922411
17	6	0	0.182172	-0.985230	0.467874
18	1	0	0.332584	-1.677977	1.292459
19	6	0	1.189563	-1.049644	-0.521033
20	1	0	1.022783	-0.915480	-1.578520
21	6	0	-1.239743	-0.827178	0.057864
22	6	0	-2.246323	-1.391668	0.860985
23	6	0	-1.624393	-0.142450	-1.108297
24	6	0	-3.593086	-1.281528	0.510305
25	1	0	-1.968421	-1.928688	1.765409
26	6	0	-2.970969	-0.032652	-1.461226
27	1	0	-0.871662	0.308585	-1.749009
28	6	0	-3.961637	-0.601258	-0.654324
29	1	0	-4.352966	-1.732397	1.143370
30	1	0	-3.246140	0.496306	-2.370217
31	1	0	-5.008943	-0.517290	-0.931967
32	7	0	2.393456	-1.731053	-0.241665
33	8	0	2.700174	-1.958579	0.951339
34	8	0	3.127609	-2.052690	-1.198763

B3LYP/6-311G(d)

TS endo

Center Number	Atomic Number	Atomic Type	Coordinates (Angstroms) X	Y	Z
1	6	0	-2.366493	0.603908	0.974851
2	6	0	-1.646167	1.611387	0.277232
3	6	0	-1.670830	-0.347871	1.696755
4	1	0	-0.714270	-0.136196	2.153241
5	1	0	-2.193008	-1.203040	2.112423
6	6	0	-0.254504	1.640858	0.351107
7	1	0	0.285691	2.381067	-0.229816
8	1	0	0.250057	1.358918	1.264934
9	6	0	-3.846355	0.420555	0.727739
10	1	0	-4.051793	0.189415	-0.321392
11	1	0	-4.249644	-0.391032	1.334486
12	1	0	-4.405763	1.327785	0.975844
13	6	0	-2.360581	2.492873	-0.716672
14	1	0	-3.131465	3.094751	-0.224461
15	1	0	-1.669802	3.180319	-1.206457
16	1	0	-2.863341	1.905797	-1.490492
17	6	0	-0.343803	-1.231433	-0.001382
18	1	0	0.041910	-2.008654	0.638665
19	6	0	0.433979	-0.187394	-0.525108
20	1	0	0.145730	0.137745	-1.516339
21	6	0	1.878208	-0.084440	-0.217171
22	6	0	2.733906	0.517741	-1.152080
23	6	0	2.434703	-0.553971	0.983213
24	6	0	4.098460	0.634069	-0.905075
25	1	0	2.322408	0.890085	-2.085552
26	6	0	3.798777	-0.435656	1.231418
27	1	0	1.802533	-1.018361	1.733366
28	6	0	4.637757	0.157505	0.288619
29	1	0	4.741556	1.096160	-1.647082
30	1	0	4.208160	-0.808883	2.164608
31	1	0	5.701188	0.247775	0.483451
32	7	0	-1.472847	-1.686365	-0.759449
33	8	0	-1.950728	-0.933246	-1.616733
34	8	0	-1.933341	-2.800238	-0.496264

B3LYP/6-311G(d)

TS exo

Center Number	Atomic Number	Atomic Type	Coordinates (Angstroms) X	Y	Z
1	6	0	0.391367	1.752646	0.985532
2	6	0	1.243810	1.997969	-0.126116
3	6	0	0.575707	0.606482	1.760763
4	1	0	1.565028	0.202920	1.936047
5	1	0	-0.121204	0.418644	2.571418
6	6	0	2.307252	1.153057	-0.367060
7	1	0	2.882953	1.241593	-1.282198
8	1	0	2.809388	0.632726	0.435816
9	6	0	-0.850394	2.586200	1.176226
10	1	0	-1.388634	2.298659	2.079540
11	1	0	-0.602717	3.649152	1.257989
12	1	0	-1.540893	2.487541	0.331502
13	6	0	0.883244	3.062683	-1.138679
14	1	0	-0.133946	2.950170	-1.525538
15	1	0	0.938877	4.061147	-0.692735
16	1	0	1.570494	3.047896	-1.985722
17	6	0	0.210203	-0.995308	0.451966
18	1	0	0.389590	-1.667187	1.283432
19	6	0	1.232891	-0.976124	-0.510554
20	1	0	1.066264	-0.880770	-1.570397
21	6	0	-1.208438	-0.860464	0.050871
22	6	0	-2.204220	-1.420650	0.865633
23	6	0	-1.606490	-0.197114	-1.119724
24	6	0	-3.549180	-1.326850	0.522602
25	1	0	-1.916685	-1.941703	1.774118
26	6	0	-2.952445	-0.102214	-1.463026
27	1	0	-0.863551	0.248018	-1.772527
28	6	0	-3.930074	-0.665813	-0.644436
29	1	0	-4.300622	-1.774319	1.165066
30	1	0	-3.237982	0.411050	-2.375667
31	1	0	-4.978182	-0.593538	-0.915165
32	7	0	2.452792	-1.664623	-0.227221
33	8	0	2.753476	-1.883732	0.956067
34	8	0	3.175633	-1.969597	-1.179618

B3LYP/6-311+G(d)

TS endo

Center Number	Atomic Number	Atomic Type	Coordinates (Angstroms) X	Y	Z
1	6	0	-2.406539	0.626927	0.957604
2	6	0	-1.644981	1.594083	0.240906
3	6	0	-1.757029	-0.306615	1.735090
4	1	0	-0.784479	-0.125904	2.168744
5	1	0	-2.304753	-1.137664	2.166395
6	6	0	-0.249580	1.590815	0.339630
7	1	0	0.307388	2.326835	-0.230944
8	1	0	0.219700	1.338890	1.281398
9	6	0	-3.889356	0.489510	0.700025
10	1	0	-4.100361	0.284504	-0.352898
11	1	0	-4.318320	-0.319917	1.291906
12	1	0	-4.421426	1.409095	0.963762
13	6	0	-2.318170	2.469117	-0.783900
14	1	0	-3.084633	3.097473	-0.318115
15	1	0	-1.603346	3.129780	-1.275938
16	1	0	-2.822447	1.877178	-1.553444
17	6	0	-0.322226	-1.251013	0.003630
18	1	0	0.064806	-1.999221	0.676158
19	6	0	0.427528	-0.173776	-0.504079
20	1	0	0.151114	0.125422	-1.507698
21	6	0	1.878393	-0.069534	-0.201236
22	6	0	2.734369	0.495458	-1.157937
23	6	0	2.433219	-0.502747	1.012759
24	6	0	4.101480	0.610815	-0.918085
25	1	0	2.324068	0.839065	-2.102919
26	6	0	3.799339	-0.385762	1.254312
27	1	0	1.800212	-0.937676	1.779851
28	6	0	4.640327	0.170421	0.289953
29	1	0	4.745689	1.043460	-1.676673
30	1	0	4.208454	-0.730664	2.198438
31	1	0	5.704834	0.259717	0.479428
32	7	0	-1.439481	-1.719575	-0.738532
33	8	0	-1.936469	-0.975818	-1.598908
34	8	0	-1.884877	-2.847873	-0.479811

B3LYP/6-311+G(d)

TS exo

Center Number	Atomic Number	Atomic Type	Coordinates (Angstroms) X	Y	Z
1	6	0	0.408694	1.738893	0.980673
2	6	0	1.276893	2.008979	-0.116325
3	6	0	0.582411	0.573773	1.736654
4	1	0	1.574883	0.183080	1.927142
5	1	0	-0.116152	0.388776	2.546763
6	6	0	2.350864	1.182273	-0.354833
7	1	0	2.938079	1.288655	-1.260686
8	1	0	2.824681	0.618292	0.435120
9	6	0	-0.831605	2.570624	1.175551
10	1	0	-1.378382	2.269611	2.069219
11	1	0	-0.581403	3.631541	1.275244
12	1	0	-1.514821	2.488859	0.322771
13	6	0	0.927047	3.095624	-1.109186
14	1	0	-0.082895	2.986039	-1.515231
15	1	0	0.969429	4.083351	-0.638737
16	1	0	1.628396	3.104263	-1.944668
17	6	0	0.199758	-0.983922	0.461142
18	1	0	0.363236	-1.668788	1.286168
19	6	0	1.214315	-1.010855	-0.514813
20	1	0	1.048941	-0.889633	-1.572092
21	6	0	-1.220514	-0.842529	0.053443
22	6	0	-2.219812	-1.400726	0.864900
23	6	0	-1.612267	-0.175984	-1.117099
24	6	0	-3.564398	-1.302719	0.517688
25	1	0	-1.937723	-1.924507	1.773571
26	6	0	-2.957297	-0.077078	-1.465268
27	1	0	-0.866848	0.270217	-1.766579
28	6	0	-3.939579	-0.639376	-0.650380
29	1	0	-4.318975	-1.749013	1.157280
30	1	0	-3.238409	0.438186	-2.378170
31	1	0	-4.986557	-0.564262	-0.924791
32	7	0	2.423764	-1.697552	-0.235372
33	8	0	2.724804	-1.928888	0.949221
34	8	0	3.152836	-2.007819	-1.188922

ωB97xD/6-31G(d)

TS endo

Center Number	Atomic Number	Atomic Type	Coordinates (Angstroms) X	Y	Z
1	6	0	-2.313154	0.577822	1.017573
2	6	0	-1.599919	1.579621	0.296179
3	6	0	-1.617566	-0.368074	1.730121
4	1	0	-0.626617	-0.186849	2.127545
5	1	0	-2.137604	-1.221328	2.156791
6	6	0	-0.214473	1.611653	0.348533
7	1	0	0.318700	2.352855	-0.241800
8	1	0	0.310439	1.310043	1.247611
9	6	0	-3.794880	0.403139	0.788178
10	1	0	-4.002248	0.152886	-0.258267
11	1	0	-4.197449	-0.396971	1.413907
12	1	0	-4.342554	1.322613	1.023695
13	6	0	-2.333996	2.444596	-0.694856
14	1	0	-3.104955	3.042078	-0.194199
15	1	0	-1.652299	3.133244	-1.200011
16	1	0	-2.836177	1.836940	-1.455043
17	6	0	-0.336825	-1.243710	-0.084474
18	1	0	0.033376	-2.044419	0.538209
19	6	0	0.431725	-0.191902	-0.575881
20	1	0	0.142396	0.179064	-1.553024
21	6	0	1.873898	-0.100859	-0.246834
22	6	0	2.746231	0.499200	-1.160743
23	6	0	2.394185	-0.573159	0.964279
24	6	0	4.104659	0.608064	-0.882779
25	1	0	2.353757	0.878641	-2.100780
26	6	0	3.751696	-0.463319	1.243577
27	1	0	1.736199	-1.027667	1.700660
28	6	0	4.612788	0.125941	0.320527
29	1	0	4.767030	1.070683	-1.608236
30	1	0	4.137931	-0.837470	2.186914
31	1	0	5.672609	0.211237	0.539808
32	7	0	-1.518622	-1.606633	-0.792157
33	8	0	-1.983283	-0.805449	-1.608013
34	8	0	-2.037767	-2.692453	-0.536872

ωB97xD/6-31G(d)

TS exo

Center Number	Atomic Number	Atomic Type	Coordinates (Angstroms) X	Y	Z
1	6	0	0.106627	1.609393	1.065616
2	6	0	0.828543	2.044335	-0.083528
3	6	0	0.560215	0.522226	1.797557
4	1	0	1.615935	0.288724	1.893307
5	1	0	-0.049240	0.170114	2.626740
6	6	0	2.034181	1.461951	-0.389355
7	1	0	2.531021	1.684575	-1.329654
8	1	0	2.669862	1.034548	0.376630
9	6	0	-1.280683	2.140426	1.314937
10	1	0	-1.683963	1.767762	2.259202
11	1	0	-1.285035	3.235562	1.348695
12	1	0	-1.966698	1.835854	0.513698
13	6	0	0.178672	3.002132	-1.055050
14	1	0	-0.817299	2.665724	-1.363997
15	1	0	0.054382	3.991941	-0.599767
16	1	0	0.791454	3.126566	-1.951678
17	6	0	0.398537	-1.050677	0.396982
18	1	0	0.641143	-1.722251	1.215119
19	6	0	1.401157	-0.853402	-0.550100
20	1	0	1.238700	-0.747216	-1.610920
21	6	0	-1.024090	-1.001964	-0.006659
22	6	0	-1.990437	-1.600273	0.809338
23	6	0	-1.446560	-0.333818	-1.161840
24	6	0	-3.339644	-1.536272	0.481602
25	1	0	-1.677726	-2.117802	1.712854
26	6	0	-2.796933	-0.268458	-1.490666
27	1	0	-0.721293	0.152982	-1.806636
28	6	0	-3.748894	-0.868216	-0.670348
29	1	0	-4.073492	-2.009535	1.126975
30	1	0	-3.104944	0.254200	-2.391369
31	1	0	-4.802392	-0.816864	-0.927379
32	7	0	2.720450	-1.297307	-0.252402
33	8	0	3.032446	-1.483965	0.929109
34	8	0	3.506590	-1.430789	-1.188598

ωB97xD /6-31+G(d)

TS endo

Center Number	Atomic Number	Atomic Type	Coordinates (Angstroms) X	Y	Z
1	6	0	-2.355248	0.590933	1.007084
2	6	0	-1.607561	1.554681	0.260317
3	6	0	-1.695971	-0.331652	1.776116
4	1	0	-0.690286	-0.178817	2.146381
5	1	0	-2.236425	-1.162305	2.221975
6	6	0	-0.217067	1.563701	0.334683
7	1	0	0.327251	2.303942	-0.247335
8	1	0	0.275791	1.295671	1.262961
9	6	0	-3.839133	0.450495	0.767505
10	1	0	-4.046498	0.188580	-0.276090
11	1	0	-4.266762	-0.328508	1.403466
12	1	0	-4.363198	1.388316	0.984974
13	6	0	-2.309460	2.412116	-0.757533
14	1	0	-3.051547	3.057759	-0.272163
15	1	0	-1.603304	3.054067	-1.290046
16	1	0	-2.843565	1.800442	-1.492606
17	6	0	-0.309763	-1.262156	-0.091514
18	1	0	0.063194	-2.041129	0.556546
19	6	0	0.432049	-0.173436	-0.558974
20	1	0	0.157273	0.177630	-1.548963
21	6	0	1.879681	-0.082651	-0.231543
22	6	0	2.756198	0.478229	-1.166300
23	6	0	2.395733	-0.522241	0.993923
24	6	0	4.117866	0.582634	-0.892939
25	1	0	2.367379	0.831098	-2.118626
26	6	0	3.755770	-0.417077	1.269201
27	1	0	1.734184	-0.946493	1.745784
28	6	0	4.622688	0.134054	0.325837
29	1	0	4.784244	1.014506	-1.634052
30	1	0	4.139526	-0.765010	2.223918
31	1	0	5.683949	0.215410	0.541521
32	7	0	-1.486705	-1.627282	-0.779602
33	8	0	-1.971641	-0.822153	-1.586451
34	8	0	-2.000730	-2.725341	-0.534251

ωB97xD /6-31+G(d)

TS exo

Center Number	Atomic Number	Atomic Type	Coordinates (Angstroms) X	Y	Z
1	6	0	0.133431	1.606674	1.061698
2	6	0	0.864284	2.060935	-0.078130
3	6	0	0.575766	0.498224	1.776490
4	1	0	1.633044	0.274409	1.887180
5	1	0	-0.036330	0.152224	2.606748
6	6	0	2.074463	1.491672	-0.382606
7	1	0	2.575149	1.722621	-1.319064
8	1	0	2.695383	1.023314	0.371399
9	6	0	-1.248359	2.145533	1.318241
10	1	0	-1.652496	1.770020	2.261091
11	1	0	-1.244192	3.240594	1.357040
12	1	0	-1.938912	1.851382	0.516286
13	6	0	0.220679	3.037976	-1.035238
14	1	0	-0.774471	2.709020	-1.355363
15	1	0	0.096909	4.019806	-0.562748
16	1	0	0.838551	3.176726	-1.926462
17	6	0	0.382592	-1.037805	0.410846
18	1	0	0.609794	-1.724150	1.222533
19	6	0	1.384131	-0.889507	-0.552468
20	1	0	1.218089	-0.758290	-1.610066
21	6	0	-1.041848	-0.983242	0.000323
22	6	0	-2.011441	-1.583135	0.812232
23	6	0	-1.459324	-0.314551	-1.156770
24	6	0	-3.360943	-1.519853	0.478391
25	1	0	-1.703442	-2.102292	1.716918
26	6	0	-2.808987	-0.251367	-1.493294
27	1	0	-0.732425	0.175411	-1.798080
28	6	0	-3.765476	-0.852571	-0.677183
29	1	0	-4.097552	-1.994559	1.120179
30	1	0	-3.113134	0.271495	-2.395664
31	1	0	-4.818018	-0.802949	-0.940068
32	7	0	2.693335	-1.338172	-0.260697
33	8	0	3.007626	-1.543214	0.920454
34	8	0	3.486986	-1.468221	-1.198227

ωB97xD /6-311G(d)

TS endo

Center Number	Atomic Number	Atomic Type	Coordinates (Angstroms) X	Y	Z
1	6	0	-2.294695	0.573724	1.024605
2	6	0	-1.595321	1.583963	0.308590
3	6	0	-1.584641	-0.376737	1.715631
4	1	0	-0.599222	-0.185560	2.118350
5	1	0	-2.094486	-1.235708	2.139772
6	6	0	-0.212093	1.620795	0.349002
7	1	0	0.314953	2.361147	-0.245027
8	1	0	0.322706	1.318507	1.239767
9	6	0	-3.773664	0.385024	0.800538
10	1	0	-3.984295	0.132895	-0.242876
11	1	0	-4.168174	-0.416121	1.426517
12	1	0	-4.329753	1.297021	1.035714
13	6	0	-2.340661	2.447817	-0.671910
14	1	0	-3.107819	3.041156	-0.164808
15	1	0	-1.669220	3.140072	-1.181478
16	1	0	-2.848463	1.843067	-1.427837
17	6	0	-0.346930	-1.234296	-0.084404
18	1	0	0.021065	-2.043627	0.526015
19	6	0	0.429849	-0.193293	-0.578998
20	1	0	0.141635	0.183389	-1.552241
21	6	0	1.869950	-0.106069	-0.250485
22	6	0	2.738381	0.511963	-1.152931
23	6	0	2.393395	-0.597185	0.949251
24	6	0	4.094387	0.621451	-0.874921
25	1	0	2.344787	0.905780	-2.085350
26	6	0	3.748559	-0.486403	1.228821
27	1	0	1.741725	-1.070039	1.677815
28	6	0	4.605073	0.121810	0.317478
29	1	0	4.753783	1.098421	-1.592254
30	1	0	4.137218	-0.875880	2.163778
31	1	0	5.663740	0.207558	0.537090
32	7	0	-1.531151	-1.596111	-0.800488
33	8	0	-1.984872	-0.799831	-1.616028
34	8	0	-2.054085	-2.670754	-0.540362

ωB97xD /6-311G(d)

TS exo

Center Number	Atomic Number	Atomic Type	Coordinates (Angstroms) X	Y	Z
1	6	0	0.081228	1.603113	1.068259
2	6	0	0.790474	2.040724	-0.083429
3	6	0	0.553754	0.527920	1.801689
4	1	0	1.611139	0.313381	1.898364
5	1	0	-0.046200	0.166653	2.631807
6	6	0	2.000521	1.470445	-0.390340
7	1	0	2.492013	1.692668	-1.332000
8	1	0	2.649520	1.072918	0.378697
9	6	0	-1.312890	2.110369	1.318371
10	1	0	-1.699856	1.755530	2.273910
11	1	0	-1.342140	3.203491	1.325024
12	1	0	-2.000657	1.773572	0.534378
13	6	0	0.125191	2.981564	-1.057875
14	1	0	-0.875991	2.646580	-1.342896
15	1	0	0.012603	3.977867	-0.618640
16	1	0	0.719674	3.091540	-1.966271
17	6	0	0.414767	-1.046107	0.394408
18	1	0	0.663914	-1.715316	1.210031
19	6	0	1.416152	-0.826850	-0.545365
20	1	0	1.256173	-0.727612	-1.605815
21	6	0	-1.005945	-1.009600	-0.010503
22	6	0	-1.968932	-1.599980	0.811855
23	6	0	-1.431070	-0.356847	-1.170461
24	6	0	-3.316332	-1.542362	0.486090
25	1	0	-1.655018	-2.106603	1.719845
26	6	0	-2.779905	-0.298183	-1.497666
27	1	0	-0.710541	0.123870	-1.823010
28	6	0	-3.728071	-0.888727	-0.670829
29	1	0	-4.047900	-2.009622	1.136711
30	1	0	-3.090021	0.213184	-2.402897
31	1	0	-4.781035	-0.842473	-0.926753
32	7	0	2.742699	-1.266902	-0.249284
33	8	0	3.051626	-1.464242	0.923407
34	8	0	3.524846	-1.380032	-1.182248

ωB97xD /6-311+G(d)

TS endo

Center Number	Atomic Number	Atomic Type	Coordinates (Angstroms) X	Y	Z
1	6	0	-2.327655	0.596436	1.007858
2	6	0	-1.596959	1.572801	0.271269
3	6	0	-1.651105	-0.336183	1.748410
4	1	0	-0.655564	-0.165954	2.133966
5	1	0	-2.180342	-1.174274	2.190306
6	6	0	-0.211066	1.588464	0.333514
7	1	0	0.330228	2.322355	-0.255919
8	1	0	0.296578	1.310977	1.248365
9	6	0	-3.809162	0.441672	0.776748
10	1	0	-4.025286	0.205215	-0.268807
11	1	0	-4.223078	-0.356041	1.394662
12	1	0	-4.344098	1.364316	1.020693
13	6	0	-2.311216	2.426311	-0.738758
14	1	0	-3.076530	3.042086	-0.255940
15	1	0	-1.621130	3.095978	-1.253555
16	1	0	-2.819187	1.813436	-1.488243
17	6	0	-0.328861	-1.248642	-0.074345
18	1	0	0.044403	-2.033117	0.564291
19	6	0	0.426305	-0.184184	-0.561660
20	1	0	0.146619	0.168328	-1.547154
21	6	0	1.870675	-0.093384	-0.237026
22	6	0	2.741921	0.482909	-1.163422
23	6	0	2.390492	-0.543579	0.979877
24	6	0	4.100110	0.591144	-0.891070
25	1	0	2.351029	0.844931	-2.109925
26	6	0	3.747255	-0.434162	1.254070
27	1	0	1.734893	-0.981275	1.727127
28	6	0	4.608021	0.131971	0.318896
29	1	0	4.762608	1.035167	-1.626662
30	1	0	4.133932	-0.791235	2.202826
31	1	0	5.667851	0.216612	0.534051
32	7	0	-1.503030	-1.628521	-0.777502
33	8	0	-1.978171	-0.840426	-1.593011
34	8	0	-2.008610	-2.717986	-0.521205

ωB97xD /6-311+G(d)

TS exo

Center Number	Atomic Number	Atomic Type	Coordinates (Angstroms) X	Y	Z
1	6	0	0.102425	1.601145	1.063970
2	6	0	0.822326	2.054450	-0.077584
3	6	0	0.563476	0.509934	1.785316
4	1	0	1.621100	0.299270	1.893649
5	1	0	-0.041184	0.152846	2.613991
6	6	0	2.034142	1.492542	-0.382067
7	1	0	2.531577	1.724517	-1.318300
8	1	0	2.668524	1.057671	0.378526
9	6	0	-1.286681	2.117250	1.316603
10	1	0	-1.685068	1.744116	2.260372
11	1	0	-1.302840	3.210330	1.347698
12	1	0	-1.972509	1.808381	0.519422
13	6	0	0.165451	3.012187	-1.041147
14	1	0	-0.828723	2.675881	-1.348638
15	1	0	0.038343	3.997856	-0.582442
16	1	0	0.772937	3.145940	-1.937644
17	6	0	0.401252	-1.035669	0.403501
18	1	0	0.639247	-1.715973	1.214024
19	6	0	1.402243	-0.854570	-0.548718
20	1	0	1.238780	-0.736844	-1.606710
21	6	0	-1.020886	-0.994517	-0.005386
22	6	0	-1.984759	-1.592094	0.810468
23	6	0	-1.443029	-0.334956	-1.162487
24	6	0	-3.331931	-1.535522	0.480239
25	1	0	-1.673521	-2.104296	1.716417
26	6	0	-2.791030	-0.277695	-1.494593
27	1	0	-0.721575	0.154185	-1.808099
28	6	0	-3.741001	-0.876340	-0.674793
29	1	0	-4.064705	-2.008463	1.125617
30	1	0	-3.099332	0.239039	-2.397567
31	1	0	-4.793250	-0.831390	-0.934536
32	7	0	2.719151	-1.300993	-0.256211
33	8	0	3.030004	-1.506079	0.917174
34	8	0	3.505764	-1.421795	-1.189951

M062x/6-31G(d)

TS endo

Center Number	Atomic Number	Atomic Type	Coordinates (Angstroms) X	Y	Z
1	6	0	-2.253650	0.487475	1.061994
2	6	0	-1.601707	1.555275	0.380380
3	6	0	-1.492689	-0.482729	1.675142
4	1	0	-0.504147	-0.276050	2.069885
5	1	0	-1.970701	-1.377211	2.066470
6	6	0	-0.220383	1.632888	0.392869
7	1	0	0.276946	2.404671	-0.189566
8	1	0	0.352069	1.276374	1.241054
9	6	0	-3.730460	0.258825	0.854076
10	1	0	-3.942359	0.025088	-0.196068
11	1	0	-4.087616	-0.570912	1.467483
12	1	0	-4.309485	1.150333	1.117318
13	6	0	-2.403735	2.445617	-0.533630
14	1	0	-3.151139	3.011193	0.034496
15	1	0	-1.759362	3.162854	-1.046560
16	1	0	-2.940077	1.859315	-1.286555
17	6	0	-0.345795	-1.219743	-0.163417
18	1	0	0.012803	-2.081162	0.381381
19	6	0	0.445588	-0.176859	-0.630076
20	1	0	0.149400	0.262880	-1.575823
21	6	0	1.879123	-0.106087	-0.282282
22	6	0	2.757994	0.578220	-1.130267
23	6	0	2.388484	-0.677012	0.891881
24	6	0	4.111915	0.672072	-0.826582
25	1	0	2.370893	1.033663	-2.038264
26	6	0	3.742052	-0.581489	1.196587
27	1	0	1.724363	-1.199843	1.575939
28	6	0	4.609449	0.091219	0.337955
29	1	0	4.779501	1.199792	-1.500568
30	1	0	4.120293	-1.032389	2.108743
31	1	0	5.665484	0.164687	0.577114
32	7	0	-1.566765	-1.492431	-0.866077
33	8	0	-2.000064	-0.630288	-1.625797
34	8	0	-2.142013	-2.548750	-0.635667

M062x/6-31G(d)

TS exo

Center Number	Atomic Number	Atomic Type	Coordinates (Angstroms) X	Y	Z
1	6	0	0.035873	1.563389	1.092421
2	6	0	0.702897	2.008654	-0.085152
3	6	0	0.567107	0.521273	1.830435
4	1	0	1.632286	0.318738	1.877038
5	1	0	-0.005355	0.133981	2.670363
6	6	0	1.923622	1.464260	-0.415402
7	1	0	2.387697	1.698452	-1.370092
8	1	0	2.602859	1.107429	0.351303
9	6	0	-1.373703	2.021717	1.363404
10	1	0	-1.725187	1.660451	2.332014
11	1	0	-1.441828	3.114794	1.358348
12	1	0	-2.060916	1.647420	0.592687
13	6	0	-0.015361	2.923306	-1.049204
14	1	0	-1.025356	2.569013	-1.281291
15	1	0	-0.118372	3.927993	-0.622655
16	1	0	0.541846	3.017395	-1.984440
17	6	0	0.451807	-1.080454	0.376711
18	1	0	0.720628	-1.734835	1.200352
19	6	0	1.440629	-0.801972	-0.559777
20	1	0	1.282238	-0.721763	-1.623891
21	6	0	-0.967281	-1.041078	-0.022197
22	6	0	-1.935935	-1.631458	0.799208
23	6	0	-1.387322	-0.372189	-1.179287
24	6	0	-3.285162	-1.560015	0.473447
25	1	0	-1.621689	-2.146116	1.704031
26	6	0	-2.739150	-0.299741	-1.505385
27	1	0	-0.658592	0.107376	-1.826598
28	6	0	-3.692834	-0.891921	-0.680753
29	1	0	-4.020940	-2.026390	1.121150
30	1	0	-3.046358	0.221965	-2.406778
31	1	0	-4.746297	-0.835197	-0.935448
32	7	0	2.788486	-1.175792	-0.252228
33	8	0	3.095036	-1.347890	0.926746
34	8	0	3.584065	-1.248709	-1.179411

M062x /6-31+G(d)

TS endo

Center Number	Atomic Number	Atomic Type	Coordinates (Angstroms) X	Y	Z
1	6	0	-2.277924	0.523842	1.044157
2	6	0	-1.603997	1.566536	0.342027
3	6	0	-1.535502	-0.437567	1.694910
4	1	0	-0.545448	-0.234591	2.087562
5	1	0	-2.026930	-1.317114	2.104505
6	6	0	-0.219009	1.628039	0.369585
7	1	0	0.291604	2.383537	-0.223370
8	1	0	0.334956	1.294663	1.239805
9	6	0	-3.759428	0.321804	0.840011
10	1	0	-3.991141	0.135277	-0.215220
11	1	0	-4.121621	-0.527202	1.424189
12	1	0	-4.321818	1.210067	1.149311
13	6	0	-2.382382	2.440046	-0.607610
14	1	0	-3.155504	3.002809	-0.071608
15	1	0	-1.727939	3.158723	-1.106231
16	1	0	-2.886980	1.839723	-1.372714
17	6	0	-0.342009	-1.229155	-0.126327
18	1	0	0.025103	-2.067440	0.448726
19	6	0	0.438935	-0.180806	-0.608057
20	1	0	0.146366	0.230681	-1.568607
21	6	0	1.875919	-0.101528	-0.266907
22	6	0	2.753200	0.547483	-1.145132
23	6	0	2.389914	-0.633734	0.924182
24	6	0	4.110838	0.647888	-0.851832
25	1	0	2.363952	0.971397	-2.067997
26	6	0	3.746839	-0.532101	1.218825
27	1	0	1.729190	-1.130317	1.631400
28	6	0	4.613206	0.107024	0.331304
29	1	0	4.776834	1.148199	-1.548680
30	1	0	4.128378	-0.952285	2.144737
31	1	0	5.671231	0.184890	0.562302
32	7	0	-1.545223	-1.541766	-0.835689
33	8	0	-2.002428	-0.697268	-1.603040
34	8	0	-2.088231	-2.620892	-0.612347

M062x /6-31+G(d)

TS exo

Center Number	Atomic Number	Atomic Type	Coordinates (Angstroms) X	Y	Z
1	6	0	0.061224	1.581419	1.083358
2	6	0	0.746966	2.021677	-0.086966
3	6	0	0.569496	0.524170	1.822477
4	1	0	1.633798	0.319133	1.888739
5	1	0	-0.016359	0.147344	2.658380
6	6	0	1.964286	1.461721	-0.406203
7	1	0	2.438974	1.686886	-1.358300
8	1	0	2.628437	1.084307	0.364338
9	6	0	-1.342030	2.063164	1.346123
10	1	0	-1.702515	1.714751	2.316512
11	1	0	-1.393036	3.157322	1.331645
12	1	0	-2.033506	1.693713	0.576051
13	6	0	0.053201	2.952016	-1.054732
14	1	0	-0.961668	2.617974	-1.297191
15	1	0	-0.033708	3.957623	-0.626047
16	1	0	0.620580	3.037823	-1.985051
17	6	0	0.432560	-1.064933	0.390950
18	1	0	0.690162	-1.727304	1.212846
19	6	0	1.425255	-0.819395	-0.555358
20	1	0	1.262783	-0.727452	-1.618241
21	6	0	-0.987561	-1.022449	-0.012342
22	6	0	-1.957056	-1.613337	0.809502
23	6	0	-1.406768	-0.361770	-1.175159
24	6	0	-3.307321	-1.548123	0.479711
25	1	0	-1.644551	-2.123671	1.717992
26	6	0	-2.758930	-0.296908	-1.506700
27	1	0	-0.679105	0.117247	-1.824907
28	6	0	-3.714281	-0.888011	-0.681112
29	1	0	-4.043503	-2.014321	1.127885
30	1	0	-3.065256	0.218809	-2.412403
31	1	0	-4.767529	-0.836888	-0.939909
32	7	0	2.763189	-1.220446	-0.253690
33	8	0	3.072176	-1.404455	0.924279
34	8	0	3.559002	-1.309997	-1.184005

M062x /6-311G(d)

TS endo

Center Number	Atomic Number	Atomic Type	Coordinates (Angstroms) X	Y	Z
1	6	0	-2.245345	0.490672	1.064530
2	6	0	-1.594766	1.558285	0.385679
3	6	0	-1.483970	-0.482106	1.671269
4	1	0	-0.500640	-0.275088	2.073403
5	1	0	-1.961690	-1.375964	2.059323
6	6	0	-0.214962	1.634152	0.395231
7	1	0	0.282157	2.402015	-0.189119
8	1	0	0.358478	1.282638	1.242310
9	6	0	-3.721269	0.261093	0.855671
10	1	0	-3.936458	0.033358	-0.192745
11	1	0	-4.078224	-0.569544	1.463777
12	1	0	-4.300500	1.148411	1.122959
13	6	0	-2.396973	2.446297	-0.528764
14	1	0	-3.145004	3.009169	0.036778
15	1	0	-1.756729	3.164690	-1.040255
16	1	0	-2.931025	1.860763	-1.280672
17	6	0	-0.352044	-1.216814	-0.159310
18	1	0	0.003290	-2.080986	0.379691
19	6	0	0.443432	-0.180339	-0.627759
20	1	0	0.148842	0.260571	-1.571269
21	6	0	1.876582	-0.111446	-0.282345
22	6	0	2.749747	0.585827	-1.123043
23	6	0	2.391209	-0.693745	0.881936
24	6	0	4.102287	0.682460	-0.821805
25	1	0	2.360132	1.049473	-2.023934
26	6	0	3.743530	-0.595240	1.184157
27	1	0	1.735505	-1.229982	1.560902
28	6	0	4.604769	0.091281	0.333273
29	1	0	4.765146	1.220091	-1.490188
30	1	0	4.125949	-1.055279	2.088198
31	1	0	5.659506	0.166830	0.570667
32	7	0	-1.575311	-1.490684	-0.867895
33	8	0	-1.999771	-0.636865	-1.632102
34	8	0	-2.152094	-2.537964	-0.631259

M062x /6-311G(d)

TS exo

Center Number	Atomic Number	Atomic Type	Coordinates (Angstroms) X	Y	Z
1	6	0	0.014401	1.553267	1.095605
2	6	0	0.670331	2.005114	-0.083140
3	6	0	0.561795	0.520958	1.832623
4	1	0	1.627903	0.338116	1.883512
5	1	0	-0.002364	0.125000	2.671711
6	6	0	1.897074	1.478367	-0.415817
7	1	0	2.354351	1.715246	-1.371167
8	1	0	2.588225	1.147505	0.349616
9	6	0	-1.401181	1.989182	1.368945
10	1	0	-1.736894	1.646628	2.347239
11	1	0	-1.492252	3.077733	1.338572
12	1	0	-2.087937	1.584557	0.616556
13	6	0	-0.063684	2.905882	-1.047243
14	1	0	-1.075727	2.552225	-1.258706
15	1	0	-0.158168	3.913913	-0.632540
16	1	0	0.476862	2.990123	-1.990563
17	6	0	0.466156	-1.077975	0.371784
18	1	0	0.738447	-1.732289	1.191614
19	6	0	1.453832	-0.782617	-0.557669
20	1	0	1.300026	-0.704413	-1.620808
21	6	0	-0.951827	-1.047105	-0.028705
22	6	0	-1.917697	-1.627254	0.801012
23	6	0	-1.374676	-0.392969	-1.190838
24	6	0	-3.266038	-1.559104	0.479118
25	1	0	-1.602385	-2.131231	1.709591
26	6	0	-2.725980	-0.324796	-1.513649
27	1	0	-0.651848	0.077839	-1.848098
28	6	0	-3.676454	-0.904953	-0.680429
29	1	0	-3.999318	-2.017102	1.133091
30	1	0	-3.035266	0.184969	-2.419299
31	1	0	-4.729122	-0.850916	-0.932569
32	7	0	2.808528	-1.149895	-0.249760
33	8	0	3.111885	-1.334832	0.921494
34	8	0	3.601799	-1.200745	-1.172619

M062x /6-311+G(d)

TS endo

Center Number	Atomic Number	Atomic Type	Coordinates (Angstroms) X	Y	Z
1	6	0	-2.263725	0.526950	1.045665
2	6	0	-1.593409	1.569315	0.344933
3	6	0	-1.521040	-0.436337	1.688143
4	1	0	-0.534308	-0.234288	2.083646
5	1	0	-2.011496	-1.313791	2.097723
6	6	0	-0.212064	1.631905	0.368642
7	1	0	0.297708	2.383807	-0.225525
8	1	0	0.345684	1.297765	1.233148
9	6	0	-3.744168	0.324936	0.844188
10	1	0	-3.980068	0.147014	-0.208747
11	1	0	-4.105734	-0.525372	1.421872
12	1	0	-4.305580	1.208670	1.158941
13	6	0	-2.374708	2.439587	-0.602959
14	1	0	-3.153563	2.992035	-0.069923
15	1	0	-1.728000	3.165026	-1.096053
16	1	0	-2.871071	1.839572	-1.369841
17	6	0	-0.347058	-1.225406	-0.125764
18	1	0	0.017813	-2.065391	0.444243
19	6	0	0.437357	-0.186606	-0.612365
20	1	0	0.144396	0.226740	-1.569224
21	6	0	1.872541	-0.107837	-0.271386
22	6	0	2.747973	0.547111	-1.143213
23	6	0	2.385183	-0.642625	0.916303
24	6	0	4.102315	0.648713	-0.847963
25	1	0	2.360403	0.973705	-2.063190
26	6	0	3.738835	-0.539082	1.212793
27	1	0	1.726703	-1.143323	1.619593
28	6	0	4.603202	0.104913	0.331259
29	1	0	4.767419	1.152748	-1.540028
30	1	0	4.119809	-0.961566	2.135684
31	1	0	5.658845	0.184211	0.563802
32	7	0	-1.555730	-1.538915	-0.835200
33	8	0	-2.005138	-0.704019	-1.607275
34	8	0	-2.100644	-2.607029	-0.600147

M062x /6-311+G(d)

TS exo

Center Number	Atomic Number	Atomic Type	Coordinates (Angstroms) X	Y	Z
1	6	0	0.026476	1.560863	1.090153
2	6	0	0.694519	2.015680	-0.081658
3	6	0	0.561988	0.519808	1.826467
4	1	0	1.627873	0.337537	1.888938
5	1	0	-0.010589	0.126756	2.661229
6	6	0	1.920774	1.485256	-0.407298
7	1	0	2.385407	1.723085	-1.358923
8	1	0	2.600932	1.136311	0.359868
9	6	0	-1.385944	2.007711	1.357920
10	1	0	-1.733497	1.656094	2.328865
11	1	0	-1.464597	3.097601	1.339703
12	1	0	-2.070863	1.621179	0.594494
13	6	0	-0.027721	2.925093	-1.046212
14	1	0	-1.036808	2.572543	-1.273298
15	1	0	-0.128822	3.929104	-0.623303
16	1	0	0.524655	3.018554	-1.981768
17	6	0	0.456029	-1.067526	0.380447
18	1	0	0.721629	-1.727204	1.198560
19	6	0	1.445632	-0.793542	-0.556182
20	1	0	1.288295	-0.705936	-1.618105
21	6	0	-0.962544	-1.036095	-0.022804
22	6	0	-1.927978	-1.620733	0.804513
23	6	0	-1.384783	-0.383087	-1.185856
24	6	0	-3.276398	-1.557877	0.479007
25	1	0	-1.613604	-2.124252	1.713771
26	6	0	-2.735834	-0.319938	-1.512277
27	1	0	-0.662550	0.093015	-1.840190
28	6	0	-3.686448	-0.904964	-0.681832
29	1	0	-4.009491	-2.019195	1.131006
30	1	0	-3.044954	0.189660	-2.418183
31	1	0	-4.738739	-0.855261	-0.936862
32	7	0	2.793706	-1.174961	-0.252156
33	8	0	3.099777	-1.364921	0.918724
34	8	0	3.588118	-1.238674	-1.177102
